# Structural racism as a fundamental cause of health inequities: a scoping review

**DOI:** 10.1186/s12939-025-02644-7

**Published:** 2025-10-08

**Authors:** Adnan Kisa, Sezer Kisa

**Affiliations:** 1 School of Health Sciences, Kristiania University of Applied Sciences, Oslo, Norway; 2 Department of International Health and Sustainable Development, Tulane University, New Orleans, United States of America; 3 Department of Nursing and Health Promotion, Faculty of Health Sciences, Oslo Metropolitan University, Oslo, Norway

**Keywords:** Structural racism, Health disparities, Racial health inequities, Institutional discrimination, Multisectoral interventions

## Abstract

**Background:**

Structural racism is increasingly recognized as a fundamental cause of health inequities. It operates through laws, institutional policies, and systemic practices that disproportionately disadvantage racially and ethnically minoritized populations. Although the body of evidence on structural racism and health is expanding, much of it remains fragmented across disciplines and sectors. This scoping review synthesized peer-reviewed research by examining the pathways through which structural racism affects health, the most frequent outcomes, and the interventions and policies implemented to address these disparities.

**Methods:**

The review adhered to frameworks by Arksey and O’Malley, Levac et al., and the Joanna Briggs Institute. Six databases (MEDLINE, Embase, Web of Science, CINAHL, PsycINFO, and Scopus) were searched for English-language, peer-reviewed studies published before February 15, 2025, examining structural, systemic, or institutional racism in relation to health. Two reviewers independently screened and extracted data, and findings were analyzed using thematic synthesis.

**Results:**

Eighty-three studies met the inclusion criteria, covering healthcare, housing, the criminal legal system, environmental exposures, and other intersecting sectors. Structural racism was consistently associated with adverse outcomes in maternal and infant health, cancer, cardiovascular disease, HIV care, mental health, and COVID-19. Key mechanisms included redlining, residential segregation, carceral practices, discriminatory clinical treatment, and environmental injustice. Intersectional burdens were most pronounced among Black, Indigenous, LGBQ, immigrant, and socioeconomically marginalized groups. Although some promising interventions were identified, including culturally tailored perinatal care, community health worker models, and equity-focused quality improvement, few had been rigorously evaluated or embedded in broader structural policy changes.

**Conclusion:**

Structural racism was found to operate across institutional and societal systems to perpetuate health disparities. While targeted interventions show promise, significant gaps remain in the development and implementation of scalable, evidence-based reforms. To achieve health equity, public health strategies must prioritize cross-sectoral actions for confronting and dismantling the structural conditions that maintain racial injustice. This synthesis highlights the urgent need for scalable policy reforms and structural accountability measures across sectors.

**Supplementary Information:**

The online version contains supplementary material available at 10.1186/s12939-025-02644-7.

## Introduction

Structural racism is a fundamental cause of health inequities. It operates across and between societal systems to shape life opportunities, distribute resources and risks, and ultimately determines health trajectories [1–3]. Unlike individual acts of bigotry, structural racism is embedded within laws, policies, institutional practices, and cultural norms that systematically advantage some groups while disadvantaging others [1, 4]. This system-level discrimination manifests itself in unequal access to housing, healthcare, education, employment, criminal justice, and environmental safety [5], resulting in persistent and cumulative disadvantage among racially and ethnically minoritized populations [6].

A growing body of evidence demonstrates that structural racism adversely impacts health across the life course. Residential segregation and housing discrimination are linked to preterm birth [7], infant mortality [8, 9], maternal morbidity [10], breast cancer mortality [11], cardiovascular disease [12], and exposure to environmental toxins [13]. In healthcare systems, structural inequities contribute to disparities in cardiovascular care [14], cancer outcomes [15, 16], HIV treatment [17], maternal and infant care [18, 19], pain management [20], and neurological disorders [21]. The criminal legal system also functions as a structural health determinant, with racialized policing and incarceration associated with increased risk of preterm birth [22], maternal morbidity [23], pregnancy loss [24], cardiovascular harm [25], and poor mental health [26]. Environmental racism exposes communities of color to air pollution [13], higher asthma mortality [27], and elevated breast cancer risk [28]. These exposures, compounded by psychosocial trauma and cumulative stress, contribute to elevated rates of depression, anxiety, suicidality [18, 29], and intimate partner violence [30].

The impacts of structural racism are not only widespread but also intersectional and cross-sectoral. Structural gendered racism contributes to disparities in preterm birth [31], while institutional practices such as discriminatory obstetric care disproportionately harm Black birthing people [32]. Racism intersects with gender, race/ethnicity, sexual orientation, disability status, immigration status, housing instability, economic inequality, and disenfranchisement, resulting in layered disadvantage across social systems [33, 34]. Notably, these health disparities persist even after accounting for individual-level factors such as education and income [1, 35], underscoring the systemic and structural roots of these inequities.

Despite the robustness of the evidence base, current research remains fragmented across disciplines and policy sectors. Most existing syntheses focus on specific outcomes such as maternal mortality or cancer disparities without mapping the broader structural pathways through which racism operates across domains [36]. Prior systematic reviews have provided important foundations for this field. Groos et al. (2018) cataloged quantitative approaches to measuring structural racism, while Clark et al. (2022) synthesized evidence on structural interventions affecting racial inequities [37, 38]. However, few reviews have examined the landscape of interventions and policy responses in a cross-sectoral and global context. This limits the development of coherent, multisectoral strategies to advance health equity. In this review, health disparities are conceptualized as measurable differences in health outcomes across racial and ethnic groups, whereas health equity refers to the fair and just opportunity for all individuals to achieve their highest level of health [1–3]. By explicitly applying a global lens, this study examines how structural racism operates as a determinant of health not only in the United States but also across diverse international health and policy settings.

To address these gaps, this scoping review systematically explores and synthesizes peer-reviewed research on the relationship between structural racism and health disparities. The review is guided by four core research questions: (1) How does structural racism influence health disparities? (2) What are the primary health outcomes affected by structural racism? (3) What interventions have been implemented to mitigate the effects of structural racism on health? and (4) How is structural racism manifested in healthcare systems and policies? In addressing these questions, the review aims to provide a comprehensive understanding of the mechanisms, impacts, and responses to structural racism within health systems and broader policy environments.

By synthesizing this evidence, the review addresses a critical gap in global health scholarship. It informs public health policy and clinical practice, guides future research, and supports the structural transformation urgently needed to eliminate racial and ethnic health disparities and achieve health equity. A distinctive contribution of this review is its explicit global lens that integrates U.S.-based evidence with non-U.S. studies to contextualize mechanisms across diverse health systems.

## Methodology

### Design

This scoping review’s methodology followed the framework that was developed by Arksey and O’Malley [39], refined by Levac et al. [40], and further enhanced by the Joanna Briggs Institute guidelines [41]. This approach supports the identification of key concepts, thematic domains, and gaps in the literature by synthesizing evidence across disciplines. Only original, peer-reviewed primary research studies were included to ensure methodological rigor and relevance.

### Search strategy

A comprehensive search strategy was developed in collaboration with a health sciences librarian and peer-reviewed to ensure accuracy and completeness. Searches were conducted in six major databases: MEDLINE/PubMed, Embase, Web of Science, CINAHL, PsycINFO, and Scopus. These databases were selected for their broad coverage of biomedical, public health, psychological, and social science literature relevant to structural racism and health. The search string used was (“structural racism” OR “systemic racism” OR “institutional racism” OR “racial inequality”) AND (health). The search was Limited to English-language peer-reviewed original research articles published up to February 15, 2025. No geographic restrictions were applied, enabling comparative, cross-system synthesis across health and policy settings.

### Inclusion and exclusion criteria

Studies were included if they:

- were peer-reviewed original research articles published in English;
- examined structural, systemic, or institutional racism in the context of health;
- addressed health outcomes, healthcare disparities, healthcare access, or health policy;
- employed any primary research design (qualitative, quantitative, or mixed methods); and.
- were conducted in any geographic setting.

Studies were excluded if they:

- were not peer-reviewed original research articles (e.g., opinion pieces, editorials, or grey literature);
- lacked a clear health-related component;
- focused on racism outside the health sector; or.
- did not clearly conceptualize or examine structural, systemic, or institutional racism.

### Study selection and data extraction

All search results were imported into Rayyan, where duplicate records were removed. Two independent reviewers conducted title and abstract screening, followed by full-text screening of potentially eligible studies. Discrepancies were resolved through discussion.

A standardized data extraction form was collaboratively developed and pilot-tested on a sample of studies to ensure clarity and consistency. Extracted variables included:

- article title, authors, year of publication;
- country or region, study population;
- type of racism addressed;
- health outcomes and disparities described;
- interventions implemented (if any); and.
- policy-related implications or recommendations.

### Data synthesis

Data were analyzed using thematic synthesis. Coding was conducted inductively by two researchers who independently reviewed all included articles and iteratively refined a set of themes. Studies were organized around key domains:

- pathways through which structural racism influences health;
- primary health outcomes impacted;
- types and effectiveness of interventions; and.
- how structural racism is embedded in healthcare policies, systems, and service delivery.

Both narrative and tabular formats were used to present findings clearly for researchers, policymakers, and health professionals.

### Ethical considerations

As this review was based solely on previously published, peer-reviewed literature, no ethical approval was required.

### Reporting guidelines

**Fig. 1:**
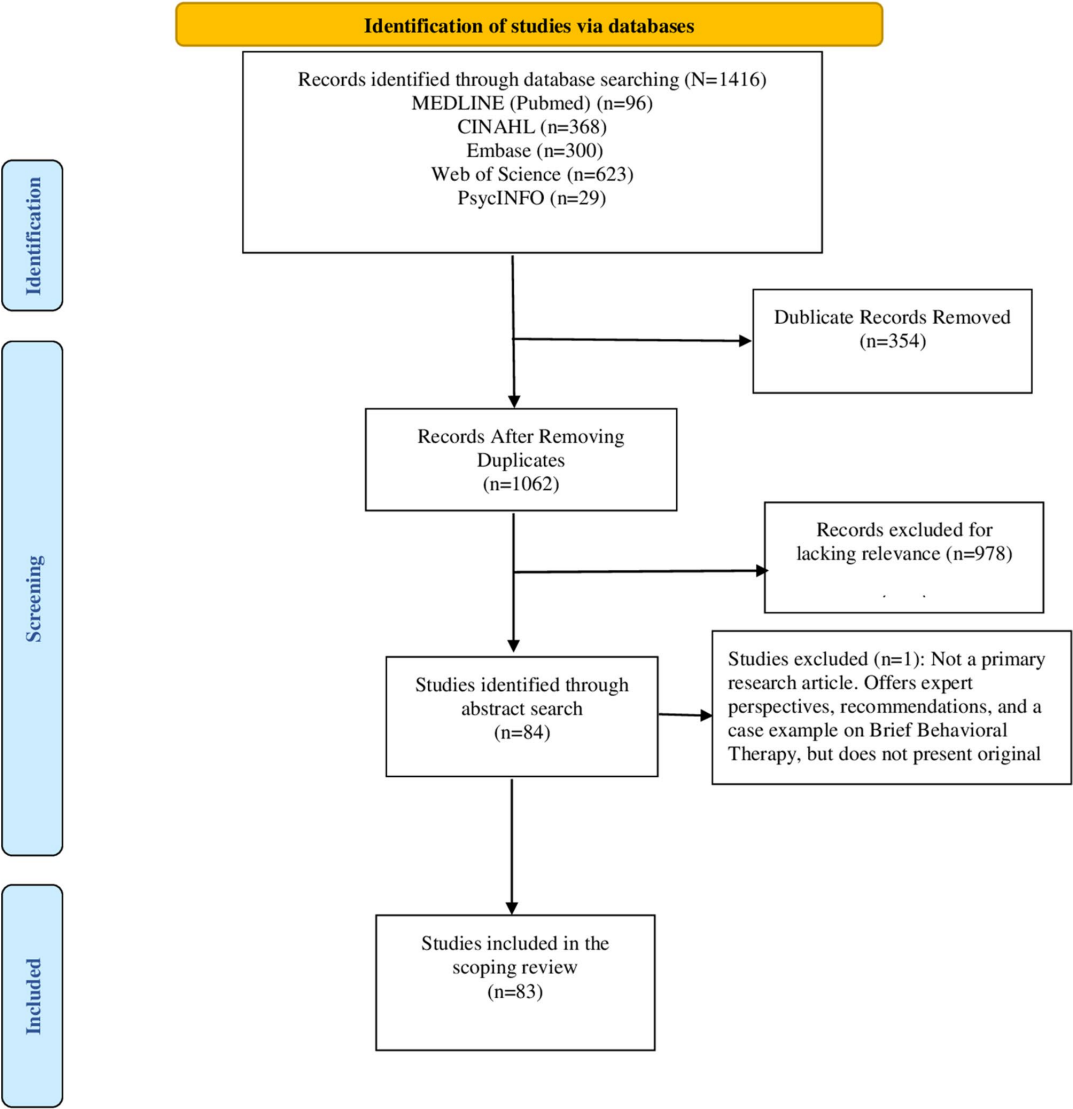
PRISMA

This review was conducted and reported in accordance with the PRISMA-ScR (Preferred Reporting Items for Systematic Reviews and Meta-Analyses extension for Scoping Reviews) checklist [42]. A full PRISMA-ScR flow diagram is provided in Fig. 1.

## Results

### Study characteristics

The 83 studies included in this review employed a diverse range of methodologies to examine structural racism and its impact on health (see Supplementary Table 1). The majority were conducted in the U.S. (*n* = 78), with additional studies from Brazil (*n* = 2), Canada (*n* = 1), the United Kingdom (UK) (*n* = 1), and Israel (*n* = 1), providing a limited but important global perspective. Observational cohort designs were the most common [11, 13, 17, 28, 43–55], alongside retrospective cohort studies addressing treatment disparities and healthcare outcomes [16, 20, 56–59]. Ecological cross-sectional designs assessed community-level exposures [8, 9, 12, 22, 27, 60–68]. Longitudinal studies provided causal insights [7, 69], and multilevel cross-sectional approaches captured contextual effects [7, 70, 71]. Mixed-methods designs evaluated community-based interventions [72, 73], while intervention studies used quasi-experimental or prospective designs [74–76]. A significant number of qualitative studies illuminated lived experiences of structural racism in healthcare and community settings [18, 19, 34, 75, 77–83].

Outside the U.S., studies examined structural racism at multiple levels of health systems, including national analyses in Brazil linking systemic inequities to excess COVID-19 mortality [64] and obesity incidence under discrimination exposure [69], a metropolitan health system study in Canada documenting systemic mistreatment and unequal access [81], a national cohort in the UK identifying elevated preterm and small-for-gestational-age risks among ethnic minority women largely explained by latent socioeconomic position [84], and hospital-level practices in Israel describing racial maternal separation affecting postpartum care [77].

Study populations were diverse across races, ethnicity, age, gender, and social identities. Many studies focused on Black American populations [17, 31, 43, 49, 51, 61, 85], with specific attention to Black maternal and infant health [9, 10, 18, 19, 23, 44, 70, 85]. Other racially and ethnically minoritized populations studied included Hispanic/Latinx [57, 64, 68], Indigenous [64], and multiracial groups [86]. LGBQ populations were addressed [34], as were immigrant and refugee youth [34]. Older adults were included in studies of felony disenfranchisement [33], and Black healthcare workers’ experiences were explored [80]. Age groups ranged from neonates [27, 49], with diverse health outcomes studied: maternal and infant health, cancer, cardiovascular disease, mental health, HIV, asthma, COVID-19, kidney disease, and surgical outcomes.

The studies covered a wide array of topics. Many studies evaluated the health impacts of residential segregation and historical redlining [11, 28, 43, 52, 58, 59, 87]. The criminal legal system’s effects on health were addressed through studies on incarceration, policing, and municipal fines [7, 22, 24, 25, 62]. Healthcare system disparities were frequently analyzed [15, 16, 21, 88]. Environmental racism and its health consequences were documented [13, 27, 65, 89]. Studies also examined economic inequality [8, 67], felony disenfranchisement [33], structural bias in public health systems [79], mental health pathways [18, 26, 81], and intersectional stigma in HIV prevention [72]. Finally, several studies focused on interventions to mitigate the effects of structural racism [72, 74, 75, 90], reflecting an emerging focus on evidence-based solutions.

#### Historical redlining and housing discrimination

A large body of evidence demonstrates that historical redlining and housing discrimination continue to shape health inequities across domains. Redlining has been linked to adverse maternal and neonatal outcomes [46, 47, 59, 91], breast cancer mortality and subtype [11, 52], HIV outcomes [43], stroke prevalence [12], cancer survival [48], and healthcare utilization [55]. Evidence further shows that redlining interacts with contemporary processes such as gentrification [10, 59] and lending bias [11], compounding disadvantage. Importantly, effects are not merely historical: contemporary housing discrimination continues to shape inequities in pregnancy-related health [58], breast cancer [52], and broader healthcare experiences [59].

#### Residential and economic segregation

Residential and economic segregation emerged as consistent drivers of racial health inequities. Associations were documented with infant and maternal outcomes [9, 49, 61, 71, 85], stillbirth [87], preterm birth [7], breast cancer mortality [45], and colorectal cancer outcomes [53]. Structural racism expressed through residential segregation also affects access to advanced medical procedures such as ventricular assist devices and kidney transplantation [14, 50], as well as postnatal healthcare outcomes among Black preterm infants [49]. The COVID-19 pandemic further highlighted the consequences of segregation on infection risk [63–65, 68].

#### Healthcare system inequities and treatment disparities

Multiple studies revealed persistent inequities in healthcare access, quality, and outcomes across clinical domains. Black and minoritized patients experienced unequal treatment in breast cancer care [15, 16, 88], cardiovascular care [14, 51], surgical outcomes [51], pain management in emergency departments [20], maternal mental health and obstetric care [18, 19], HIV care [17], and neuromyelitis optica spectrum disorder outcomes [21]. Structural racism also influences patient experience in Medicaid managed care [92], central line-associated bloodstream infection rates [74], and maternal outcomes through differential management of antepartum anemia [93]. Qualitative research underscores the cumulative effects of institutional racism across healthcare systems [77–79, 81], while frontline providers also navigate systemic biases [80].

Outside the U.S., similar patterns were observed across distinct units of analysis. In Canada (metropolitan health system level), racialized patients reported discrimination, dehumanization, negligent communication, and unequal access, contributing to distress and care avoidance [81]. In Brazil (national health system level), Black/Biracial and Indigenous patients had significantly higher COVID-19 mortality than White patients, with consistently worse outcomes in underfunded public hospitals after clinical and socioeconomic adjustment [64]. In Israel (hospital/ward level), Palestinian-Arab women experienced racial maternal separation embedded in routine maternity care, undermining equitable postpartum care and trust [77]. In the UK (national cohort), Black African, Black Caribbean, and Indian women had higher preterm birth rates, and South Asian groups had higher small-for-gestational-age rates; disparities were largely explained by latent socioeconomic position rather than single socioeconomic indicators [84].

#### Criminal legal system impacts on health

The criminal legal system consistently emerged as a critical structural determinant of health disparities. Multiple studies documented the adverse reproductive and perinatal impacts of incarceration and racialized policing. Higher county-level jail incarceration rates were associated with increased preterm birth risk [22], severe maternal morbidity [23], and adverse infant outcomes [70]. Racialized police violence, including killings, was linked to pregnancy loss [24], preterm birth [7], cardiovascular risk [25], and reductions in live birth rates [24]. Racialized policing also impacted maternal and infant outcomes through chronic stress mechanisms [26, 94]. In Israel, racial maternal separation practices reflected parallel logics of carceral control within healthcare [77]. Additionally, racially punitive municipal fiscal policies relying on fines and fees disproportionately harmed maternal health outcomes [62]. Finally, felony disenfranchisement was linked to poorer mental and physical health among older Black adults [33].

#### Maternal and infant health disparities

An extensive body of evidence linked structural racism to persistent maternal and infant health inequities. Racial and economic segregation were associated with adverse birth outcomes, including preterm birth [7, 9, 85], low birth weight [70], and small-for-gestational-age outcomes [31, 59]. Redlining further compounded disparities in maternal morbidity [10], infant mortality [8], and hypertensive disorders of pregnancy [95]. Policing and incarceration further exacerbated maternal inequities [7, 23, 24, 94]. Structural gendered racism contributed to preterm birth disparities [31], while intersectional discrimination affected postpartum blood pressure [32] and reproductive health [34]. Housing instability, including eviction, drove disparities in maternal and infant outcomes [58, 70]. Critically, studies demonstrated that socioeconomic gains did not protect Black mothers from inequities [35], underscoring the cumulative and pervasive effects of structural racism across life-course pathways.

Outside the U.S., studies have documented parallel inequities at multiple levels of the health system. In the UK (national cohort study), Black African, Black Caribbean, Indian, Pakistani, and Bangladeshi women faced elevated risks of preterm birth and small-for-gestational-age outcomes, with latent socioeconomic position explaining up to 60% of disparities—far more than traditional socioeconomic indicators [84]. In Israel (hospital and ward-level study), racial maternal separation disproportionately targeted Palestinian-Arab women in maternity care, institutionalized under the guise of cultural sensitivity and commodified healthcare, leading to inequitable postpartum experiences and reduced trust in health systems [77].

#### Mental health and stress-related pathways

Multiple studies emphasized the role of structural racism in shaping psychosocial stress and mental health outcomes. Exposure to racism and discrimination increased depressive symptoms and suicidality among Black and LGBQ adults [29], while intersectional structural oppression affected maternal stress pathways [18, 78]. Policing and racialized violence were linked to increased psychosocial distress [26, 66]. Racial discrimination was associated with worsened mental health and increased risk of intimate partner violence [30]. Internalized racism, shaped by structural barriers to healthcare access, contributes to negative health-seeking attitudes [96], compounding inequities. Furthermore, stress and bias in maternity care undermine mental health and trust among Black birthing people [19, 75], while mental health inequities were further exacerbated by the underfunding of culturally responsive services [18, 80]. Felony disenfranchisement was also linked to increased depressive symptoms among older Black adults [33], further illustrating the psychosocial consequences of structural racism.

Global studies also revealed similar psychosocial harms. In Canada, racialized healthcare users reported five clusters of mistreatment, racial/class discrimination, dehumanization, negligent communication, professional misconduct, and unequal access, which contributed to mistrust, anxiety, and delayed or avoided care [81]. In Brazil, experiences of racial discrimination intersected with educational status to increase obesity risk, demonstrating chronic stress pathways and psychosocial distress linked to systemic inequities [69].

#### Environmental racism

Excessive exposure to air pollution—including fine particulate matter and nitrogen dioxide—was strongly associated with racial residential segregation [13]. Disparities in pediatric asthma mortality were driven by structural racism through under-resourced, environmentally disadvantaged communities [27]. Environmental hazards also contributed to breast cancer disparities, with interactions between historical redlining and contemporary racialized economic segregation shaping incidence patterns of hormone receptor-negative breast cancer [28]. Maternal and infant health disparities were similarly linked to environmental injustice, with neighborhood deprivation contributing to adverse outcomes [47, 65]. The legacy of redlining continues to produce differential exposures to environmental pollutants and associated health risks [12, 59, 91]. These findings highlight the enduring health harms of spatially patterned environmental racism rooted in historical and ongoing structural inequities.

Beyond the U.S., system-level inequities intersected with environmental and social exposures: in Brazil (national health system level), marginalized racial groups experienced higher COVID-19 mortality within the public sector context of resource underfunding [64]; in a separate Brazilian cohort, perceived discrimination was associated with incident obesity among Black adults, consistent with chronic stress pathways linked to structural racism [69].

#### Cross-sectoral and intersectional impacts

Structural racism operates across and between sectors, producing cumulative and intersecting health inequities. Studies have highlighted the compounding effects of racism with other systems of oppression, such as sexism and heterosexism. Structural gendered racism shapes preterm birth disparities [31], and is linked to adverse maternal cardiovascular outcomes [32]. Intersecting racism and heterosexism within state-level criminal legal policies have increased suicidality among Black LGBQ individuals [29]. For immigrant and refugee youth, structural racism manifested through exclusionary immigration policies, language barriers, and culturally insensitive healthcare systems limit sexual and reproductive health access [34]. Structural racism was also linked to community-level violence and policing, which undermined trust in healthcare and reduced COVID vaccine uptake among Black communities [26]. Discrimination within education, housing, employment, and the criminal legal system jointly contributed to racial disparities in infant mortality [8] and COVID outcomes [66, 68]. These findings reinforce that addressing structural racism requires an intersectional, multisectoral approach that considers the complex, layered nature of systemic disadvantage.

### Structural interventions and mitigating strategies

Several studies evaluated interventions designed to mitigate the health impacts of structural racism and to promote equity. The units of analysis varied across intervention and policy studies (hospital/ward, metropolitan health system, national health system), and these are specified where applicable. Evaluated interventions were predominantly conducted in the US. Non-US studies largely offered system-level descriptions and policy recommendations rather than tested interventions. Community health worker (CHW) integrated programs demonstrated significant potential in reducing disparities in maternal outcomes. In Michigan, race-, ethnicity-, and language-concordant CHW programs improved engagement in home visiting services for high-risk birthing individuals in segregated neighborhoods [76]. These programs also reduced preterm birth and low birthweight, with larger benefits observed for Black individuals [90]. Similarly, community-based doula interventions provided culturally concordant perinatal care, buffering against systemic inequities and improving outcomes despite institutional barriers to sustainability [75].

Other community-partnered approaches targeted HIV prevention. The “Using PrEP and Doing it for Ourselves” (UPDOs) intervention, implemented through beauty salons, increased pre-exposure prophylaxis (PrEP) knowledge and trust among Black women and empowered stylist-led health advocacy [72]. In HIV care, targeted antiracist clinical strategies—including immediate antiretroviral therapy (ART) and guideline-based follow-up—substantially reduced racial disparities in HIV mortality, with Black patients benefitting the most from interventions that addressed systemic care inequities [17].

Hospital-based quality improvement initiatives also showed promise. At Seattle Children’s Hospital, equity-focused infection prevention interventions significantly reduced disparities in central line-associated bloodstream infections among minoritized racial, ethnic, and language groups [74]. In Greater Boston, community-based organizations identified gaps in social support services (housing, childcare), with structural racism limiting access to these services; enhanced coordination and investment were highlighted as critical to advancing maternal health equity [73].

At the policy level, multiple studies recommended upstream structural reforms. In Alabama, stakeholders emphasized the need for Medicaid expansion, community-based maternity models, paid parental leave, and culturally competent care to address systemic inequities in maternal health [83]. Broader reforms targeting housing and education policies [9, 67], environmental justice [65], and carceral policies [22–24] were repeatedly called for to dismantle structural drivers of health inequity. These findings emphasize the importance of both targeted health system interventions and comprehensive structural change to address the pervasive impacts of racism on health.

### Interventions evaluated across studies

While most of the 83 studies included in the review (see Supplementary Table 2) were observational, several tested interventions or offered actionable policy recommendations.

#### Housing interventions

Interventions such as community-based doula programs [75], community health worker-integrated home visiting [90], and coordinated community-based social services [73] showed potential in improving perinatal outcomes. Housing policy reforms were widely recommended [11, 52, 87, 91]. Proposed interventions include fair housing enforcement [58, 91], neighborhood reinvestment [48], environmental justice initiatives [13, 65], and integration of redlining indices into public health surveillance [11, 28, 59]. Outside the U.S., similar structural drivers were evident in Brazil, where public hospital underfunding exacerbated mortality risks for racial minorities during COVID-19 [64], emphasizing the role of national housing and social protection policies in mitigating inequities.

#### Healthcare system disparities and how to treat them

Recommendations included cultural competence training, equity-focused performance metrics [92], antiracist policies [81], and equity-driven reforms in care delivery [21, 51, 79, 81, 92]. Globally, comparable health system reforms have been called for. In Canada, racialized healthcare users described systemic mistreatment—discrimination, dehumanization, negligent communication, professional misconduct, and unequal access—prompting calls for anti-racist institutional reforms beyond cultural competence [81]. In Israel, hospital-level policies against racial maternal separation proved ineffective, with commodified care models perpetuating inequitable postpartum treatment [77]. In the U.K., findings highlighted the need to address ethnic disparities in antenatal care through broader socioeconomic and healthcare policy reforms [84].

#### Criminal legal system interventions

No direct interventions were tested, but structural reforms such as bail reform, reducing policing inequities, and addressing fiscal policies were strongly advocated [22–24, 62]. Comparable global findings reinforce this structural dimension. In Israel, maternal care practices mirrored carceral logics of control, where Palestinian-Arab women experienced racial maternal separation under institutionalized segregation [77].

#### Environmental interventions

No interventions were formally evaluated, but community-based initiatives and structural reforms were recommended to address environmental inequities [13, 27, 65, 68]. Multisector collaboration among public health, environmental justice, and urban planning stakeholders will be essential to confront the health harms of environmental racism. Brazilian evidence further highlights how underfunded public systems intersect with environmental and social exposures, producing disproportionate COVID-19 mortality among Black, Biracial, and Indigenous populations [64].

##### Maternal and infant health interventions

Community-based doula models [75], culturally specific care [80], and community health worker-integrated home visiting [90] improved engagement and outcomes. Addressing structural anemia disparities reduced maternal morbidity risk [93]. Structural reforms such as Medicaid expansion, paid parental leave, and housing reforms were consistently recommended [70, 83, 91]. Impacts of structural gendered racism were also documented [31]. Promising community-based interventions include Black-led doula care and CHW-integrated perinatal programs [75, 90]. However, scaling and sustaining these efforts will require upstream policy reforms in housing, income, and healthcare financing.

International studies similarly emphasized structural interventions. In the UK, national analyses demonstrated that ethnic disparities in preterm birth and small-for-gestational-age outcomes were strongly shaped by latent socioeconomic disadvantage [84]. In Israel, inequities in maternal care linked to racial maternal separation further highlight the necessity of hospital-level anti-racism enforcement and culturally safe perinatal care models [77].

#### Mental health, stress, and psychosocial pathways

Culturally grounded community interventions improved trust and engagement [18, 72, 80]. However, systemic underfunding remained a key barrier [72, 80]. Structural policy reforms were advocated to address mental health disparities [29, 72, 81]. At present, structural-level interventions remain limited. There is an urgent need to expand culturally responsive mental health services and address the upstream drivers of racialized trauma.

Global evidence aligns with this need. In Canada, systemic mistreatment in healthcare fostered mistrust, anxiety, and care avoidance among racialized patients [81]. In Brazil, discrimination was associated with increased obesity risk among Black adults, consistent with chronic stress and psychosocial distress pathways linked to structural inequities [69].

#### Cross-sectoral and intersectional interventions

A core insight of this review is that structural racism rarely operates in isolation. It intersects with sexism, heterosexism, xenophobia, and other systems of oppression to produce layered and compounding health inequities. Structural gendered racism [32], anti-immigrant health policy, and criminal legal bias against LGBQ communities [29] illustrate how intersecting structural forces reinforce vulnerability. Interventions that focus on a single system or identity are likely to fall short. A structural response must be both multisectoral and explicitly intersectional. Multisector interventions, including culturally concordant care [77], were proposed to address these complex drivers of health disparities [28, 34, 55, 64, 72]. Evidence from Brazil [64], the UK [84], Canada [81], and Israel [77] further reinforces that structural racism manifests differently across national contexts but consistently requires multisectoral, system-level reforms to dismantle its health impacts. A summary of interventions is presented in Supplementary Table 3.

## Discussion

This scoping review affirms that structural racism is a foundational driver of health inequities, embedded within societal systems and policies that shape population health. Across 83 studies spanning housing, healthcare, criminal justice, environmental exposure, and intersecting domains, we identified mutually reinforcing mechanisms through which structural racism operates. These findings emphasize that health inequities are not incidental or isolated but are produced and sustained by institutional practices and policy environments that systematically disadvantage racially and ethnically minoritized populations [1, 2, 5, 97].

This review offers a cross-sectoral synthesis that consolidates previously fragmented literature, providing a comprehensive mapping of how structural racism operates as a multi-domain determinant of health. The observed patterns are consistent with scholarship on fundamental social causes [98], in which multiple, replaceable mechanisms such as housing discrimination, healthcare segregation, environmental injustice, and carceral control recurrently generate inequities across settings. No formal analytic framework was applied; rather, thematic synthesis was used to organize convergent pathways across studies. The interpretation is informed by but not structured around fundamental cause theory [98], which is referenced only as conceptual background. Importantly, these pathways were evident not only in the U.S. but also internationally, including discriminatory maternity ward practices in Israel [77], ethnic disparities in perinatal outcomes in the UK [84], inequitable COVID-19 outcomes in Brazil [64], and systemic mistreatment of racialized healthcare users in Canada [81].

This synthesis is intended not only for health equity scholars but also for researchers and policymakers in adjacent fields, such as urban planning, environmental justice, and global health, who may be less familiar with structural racism as a determinant of health. By bringing together evidence from diverse sectors and national contexts, the review highlights that structural racism is a global phenomenon that undermines health through multiple institutional logics, demanding cross-sectoral and international strategies for reform.

Residential segregation has emerged as a potent determinant, associated with maternal and neonatal outcomes [59, 91], cardiovascular morbidity [12], breast and colorectal cancer mortality [28, 53], maternal depression [47], and healthcare access disparities [48]. These patterns persist despite upward mobility, indicating that structural contexts override individual-level protective factors [10, 35]. Comparable pathways were observed internationally: in the UK, latent socioeconomic position accounted for a substantial share of ethnic disparities in preterm birth and small-for-gestational-age outcomes [84], showing how structural disadvantage penetrates beyond individual socioeconomic measures.

Healthcare systems reveal entrenched inequities. Studies documented treatment disparities in oncology [15], cardiology [51], HIV care [17], neurology [21], and pain management [20]. Medicaid enrollees and racially minoritized patients frequently report diagnostic delays and perceived disrespect [82, 92], while in Canada, racialized patients described systemic mistreatment clustered into discrimination, dehumanization, negligent communication, professional misconduct, and unequal access [81]. Reforms such as equitable transplant allocation [50] are promising but remain underutilized. Interventions like equity-focused infection prevention [74] and culturally concordant perinatal care [75, 90] offer localized success, yet widespread implementation is limited.

The criminal legal system, often excluded from health equity debates, was consistently linked to adverse reproductive, cardiovascular, and mental health outcomes. Incarceration, racialized policing, and fiscal penalties were associated with preterm birth, maternal morbidity, and psychological distress [22, 23, 25, 26]. Beyond the U.S., maternity ward separation practices in Israel reflected parallel logics of carceral control, reinforcing ethnic segregation within healthcare [77]. Despite these harms, few evaluated interventions exist. Structural reforms such as bail reform, decarceration, and fiscal justice are urgently needed [62].

Structural racism also shapes mental health. Barriers to care, discriminatory encounters, and environmental stressors (e.g., police violence, pollution) elevate risks of depression, suicidality, and intimate partner violence [18, 29, 30]. Internalized racism and institutional neglect further compound harm [78]. In Canada, systemic mistreatment fostered mistrust, anxiety, and delayed care [81], while in Brazil, experiences of racial discrimination intersected with education to elevate obesity risk through psychosocial stress pathways [69]. Yet mental health systems across contexts remain underfunded and culturally disconnected [80].

Environmental racism was a pervasive and under-addressed determinant. Communities of color face disproportionate exposure to pollution and environmental degradation, contributing to asthma, breast cancer, adverse birth outcomes, and COVID-19 mortality [13, 28, 64, 65]. In Brazil, COVID-19 mortality was markedly higher among Black, Biracial, and Indigenous patients treated in underfunded public hospitals, underscoring how system-level inequities amplify environmental and health risks [64]. These findings highlight the need to integrate environmental justice into public health surveillance and policy [11, 65].

Intersectionality emerged as a core theme. Structural racism intersects with sexism, heterosexism, immigration status, and economic marginalization to produce layered harms. Examples include structural gendered racism contributing to postpartum hypertension [32], anti-immigrant health policy [34], and increased suicidality among Black LGBQ individuals [29]. These findings affirm that identity-blind approaches fall short; intersectional, multisectoral responses are essential.

Maternal health disparities remain a critical and persistent outcome of structural racism. Evidence highlights the role of obstetric racism, hospital-level discrimination, and housing instability in shaping severe maternal morbidity and mortality [19, 54, 70]. Internationally, inequitable outcomes were reported in the UK, where ethnic minority women experienced higher rates of preterm and small-for-gestational-age births linked to socioeconomic disadvantage [84], and in Israel, where Palestinian-Arab women experienced routine separation during maternity care [77]. Despite growing attention to interventions such as community health workers and Black-led doula care [19, 70, 75, 90], structural determinants such as Medicaid coverage, housing access, and income support remain under-addressed.

Yet few studies evaluated structural-level interventions. Promising models include antiracist hospital reforms [74], and CHW-integrated maternal care [90], but they remain small in scale and poorly integrated into public health systems. Only one study employed a quasi-experimental design capable of supporting causal inference [90]. The rest were observational or qualitative, such as retrospective cohort, ecological cross sectional, and interview-based studies, offering important insights into systemic patterns and lived experiences but limiting causal conclusions. Broader reforms, such as Medicaid expansion, housing reinvestment, and environmental regulatory changes, must be prioritized.

Importantly, the dominance of U.S.-based research limits global relevance. Structural racism is a global phenomenon, rooted in colonial legacies, settler institutions, and global economic marginalization [97–99]. Postcolonial contexts, Indigenous health systems, and Global South experiences are vastly underrepresented. Expanding the evidence base to include studies from Latin America, Africa, Asia, and postcolonial Europe is critical for designing globally applicable strategies.

Finally, structural racism is a political determinant of health. It reflects decisions about power, protection, and resource allocation. As Ottersen et al. [100] argue, the political origins of health inequities demand political solutions. Eliminating structural racism in health systems will require political will, cross-sectoral collaboration, and structural accountability.

### Implications for research, policy, and practice

While much of the literature remains U.S.-centric, the findings also underscore the importance of designing and evaluating interventions in global contexts. Addressing structural racism requires transformative, not incremental, change. This review identifies five key priorities. First, evaluations and scale-ups of structural interventions are urgently needed. Community designed maternal health programs, racial equity performance metrics, and environmental surveillance systems must be rigorously tested using longitudinal and mixed-method designs. For example, Brazil has proposed equity-oriented reforms in public healthcare [64] and Canada has called for anti-racist policy initiatives [81] but these remain under-evaluated. Future research should prioritize intervention testing across diverse national contexts. Second, health equity cannot be achieved solely within the healthcare sector. Housing, criminal justice, immigration, and environmental systems must be treated as core public health infrastructure, with policy integration across sectors. This is particularly evident in the UK, where socioeconomic stratification explained much of the disparity in preterm and small-for-gestational-age outcomes [84], and in Israel, where maternity ward segregation reflected structural inequities [77]. Cross-sectoral reforms are needed globally to dismantle such systemic harms. Third, the global research agenda must expand. Structural racism in health remains underexplored in low- and middle-income countries, postcolonial states, and Indigenous contexts. Studies from Brazil [64, 69] demonstrate how systemic inequities within national health systems and chronic stress pathways produce poor outcomes, but evidence from Africa, Asia, and the Middle East is sparse. Dedicated funding and publication pathways are required to support global research and ensure that strategies are not U.S.-centric. Fourth, intersectionality must be embedded in all strategies. Equity efforts should co-design interventions with affected communities, apply anti oppressive implementation frameworks, and evaluate outcomes through intersectional lenses. The Canadian evidence of layered mistreatment [81] and the Israeli documentation of ethnic segregation in maternity care [77] illustrate how structural racism rarely acts alone; it intersects with gender, immigration status, and class, demanding explicitly intersectional policy responses. Finally, structural accountability must be institutionalized. Governments and health systems should implement tools such as racial equity audits, public equity dashboards, racial impact assessments, and community led equity review boards. Without political commitment to structural change, health equity will remain aspirational. International contexts further demonstrate this need: Brazil’s COVID-19 disparities reflect underfunded public infrastructure [64], and the UK findings on perinatal disparities [84] show how latent disadvantage is embedded in socioeconomic structures. Accountability frameworks must therefore be adapted to diverse governance systems and scaled globally.

#### Limitations

This review has several Limitations. First, as a scoping review, it did not include a formal appraisal of study quality, meaning that findings from studies with varying methodological rigor were synthesized without weighing their scientific robustness. Second, most of the included studies were observational in nature such as cross-sectional and retrospective cohort designs which limits the ability to draw causal conclusions about the relationship between structural racism and health outcomes. Third, the evidence focused predominantly on the U.S.A., with limited representation from other high-income, low-income, and middle-income settings, as well as postcolonial contexts. This constrains the generalizability of findings and may overlook context-specific manifestations of structural racism in global health. Fourth, although the review identified several promising interventions, there remains a notable lack of rigorously evaluated strategies in key domains such as the criminal legal system, environmental racism, and structural determinants of mental health. Fifth, the exclusion of gray literature, such as community reports, policy briefs, and advocacy evaluations, may have omitted valuable insights into real-world interventions and grassroots innovations that are often not published in peer reviewed journals. Sixth, due to the heterogeneity of study designs, populations, and outcomes, the review did not conduct a quantitative synthesis or meta-analysis, which limits the ability to assess pooled effect sizes or compare strength across pathways. Seventh, the review only included peer reviewed research published in English, which may have excluded important studies conducted in other languages and regions. This Likely underrepresents Global South scholarship and highlights the need for targeted funding and multilingual synthesis to address this gap. Eighth, only studies published up to February 15, 2025, were included; more recent studies were not captured. Finally, the review did not analyze how the publication landscape has shifted over time, which could have provided insight into evolving research priorities and responses to major social or policy events.

## Conclusion

Building on prior reviews that cataloged measurement approaches [38] and evaluated structural interventions largely within the Organisation for Economic Co-operation and Development (OECD)/U.S. contexts [37], this scoping review extends the scholarship by applying a global lens that integrates pathways, health outcomes, and interventions across regions and sectors. The evidence demonstrates that structural racism is a fundamental driver of health inequities, operating through housing discrimination, unequal access to and quality of healthcare, the criminal legal system, environmental injustice, and chronic psychosocial stress. These effects are cumulative, intersectional, and intergenerational, shaping health trajectories well before birth and throughout the life course.

Promising interventions are beginning to emerge, particularly in community-based maternal care, antiracist healthcare delivery, and environmental health. However, their long-term effectiveness and scalability remain uncertain. Major gaps persist in areas such as criminal justice reform, housing policy, and the structural determinants of mental health. Addressing these challenges will require not only more rigorous research but also coordinated policy action across sectors.

Achieving health equity will require moving beyond fragmented, individual-level interventions toward comprehensive structural transformation. Health systems alone cannot eliminate racism. Lasting progress depends on reforms across housing, education, the criminal legal system, economic policy, and environmental governance. Without such systemic change, racial and ethnic health disparities will remain deeply rooted. Advancing this agenda must be a collective priority for public health leaders, policymakers, and researchers.

## Supplementary Information

Below is the link to the electronic supplementary material.

Supplementary Material 1.

Supplementary Material 2.

Supplementary Material 3.

## Data Availability

All data generated or analyzed during this study are included in this published article.
