# Supplementary material for: Structural racism as a fundamental cause of health inequities: a scoping review
Source: Int J Equity Health. 2025 Oct 8;24:257. doi: 10.1186/s12939-025-02644-7 (PMC12506018; doi:10.1186/s12939-025-02644-7)
Supplement: Supplementary file 1 — Supplementary Material 1. [file 12939_2025_2644_MOESM1_ESM.docx]

**Supplementary Table 1.** **Overview of Studies Examining the Health Impacts of Structural Racism Across Domains**

| **Author(s), Year, Country** | **Study Design** | **Population** | **Study Purpose** | **Key Findings** | **Relevance to Structural Racism** |
| --- | --- | --- | --- | --- | --- |
| Bassler et al., 2024, USA[41] | Observational cohort study | 1,132 adults aged ≥13 years newly diagnosed with HIV in New Orleans, Louisiana, USA (2011–2019), residing in historically redlined or non-redlined neighborhoods as defined by Home Owners’ Loan Corporation (HOLC) maps | To evaluate whether historical redlining impacts time to viral suppression among individuals with HIV | The median time to viral suppression was longer among individuals residing in historically redlined neighborhoods (193 days) compared to those in non-redlined neighborhoods (164 days). The disparity persisted despite gentrification. | Structural racism, operationalized through historical redlining, produces disparities in HIV treatment outcomes by embedding neighborhood-level disadvantage, constraining healthcare access, and perpetuating cumulative barriers to timely viral suppression. |
| Beyer et al., 2019, USA[58] | Ecological cross-sectional study | Black and White populations aged ≥25 years across the 100 largest metropolitan statistical areas (MSAs) in the United States, 2009–2013 | To examine whether mortgage discrimination or residential segregation are associated with cancer mortality disparities between Black and White populations | Mortgage discrimination was significantly correlated with larger Black–White cancer mortality disparities. Racial segregation was associated with higher disparities among Black men, driven by both higher cancer incidence and poorer survival. | Structural racism embedded in discriminatory mortgage lending and residential segregation produces disparities in cancer mortality by shaping healthcare access, neighborhood resources, and cumulative life-course exposures. |
| Bishop-Royse et al., 2021, USA [59] | Ecological cross-sectional study | 77 community areas in Chicago, Illinois, using data from 2012 to 2016 | To examine whether structural racism and economic marginalization, as measured by the Index of Concentration at the Extremes, are associated with community-level infant mortality rates | Communities with high Black concentration and economic deprivation had infant mortality rates 3.5–3.6 times higher than White-concentrated communities. The Index of Concentration at the Extremes remained a significant predictor after adjustment for confounding factors. | Structural racism, quantified through racial and economic segregation, produces disparities in infant mortality by shaping neighborhood disinvestment, differential access to healthcare and resources, and exposure to chronic stressors. |
| Bitsie et al., 2024, USA [15] | Prospective cohort study | 1,836 breast cancer patients at Kaiser Permanente Northern California (1,350 non-Hispanic White; 486 racially/ethnically minoritized) | To examine racial and ethnic discrimination in patient–provider interactions during breast cancer care | Racially and ethnically minoritized women were significantly more likely to report discrimination than non-Hispanic White women. Black women had the highest reported odds of perceived discrimination. | Structural racism and interpersonal discrimination within healthcare settings undermine patient trust, adversely affect patient–provider relationships, and contribute to disparities in breast cancer care and outcomes. |
| Boley et al., 2024, USA [20] | Retrospective cohort study | 7,309 adult patients (4,719 non-Hispanic White, 1,988 non-Hispanic Black, 602 Hispanic) presenting with undifferentiated abdominal pain to three emergency departments in the Minneapolis–St. Paul metropolitan area | To examine racial disparities in opioid administration and prescribing practices for emergency department patients presenting with abdominal pain | Non-Hispanic Black and Hispanic patients were less likely to receive opioids during emergency department visits and at discharge compared to non-Hispanic White patients. Non-Hispanic Black patients were also less likely to receive opioids prior to imaging results. | Structural racism within healthcare systems contributes to racial disparities in pain management by perpetuating biased opioid prescribing practices, reflecting implicit and structural biases in clinical decision-making. |
| Brase et al., 2021, USA [42] | Retrospective cohort study | 2,326,512 college-educated women giving birth in the United States (2015–2016), comparing non-Hispanic Black and non-Hispanic White women | To assess racial inequity in preterm delivery between non-Hispanic Black and non-Hispanic White women with college education | Non-Hispanic Black women had a 77% higher risk of preterm delivery compared to non-Hispanic White women. After adjustment for known risk factors, the risk remained 47% higher. Disparities persisted despite equivalent educational attainment. | Structural racism operates beyond individual socioeconomic attainment by producing sustained racial disparities in birth outcomes through both measured and unmeasured life-course exposures, including systemic discrimination, differential stress, and cumulative disadvantage. |
| Canales et al., 2023, USA [43] | Retrospective cohort study | 5,231 non-Hispanic Black women aged 66–90 years with stage I–IV breast cancer, diagnosed between 2007 and 2013, in the United States using SEER-Medicare data across multiple Metropolitan Statistical Areas (MSAs) | To assess the impact of local and metropolitan area isolation on all-cause and breast cancer-specific mortality | High local and metropolitan area isolation was independently associated with increased mortality. Interaction effects indicated that ethnic density in highly segregated areas may attenuate mortality risk. | Structural racism embedded in residential segregation contributes to disparities in breast cancer survival among older Black women by producing both structural barriers to care and contexts of community resilience, reflecting the complex and dual nature of segregation effects. |
| Cascino et al., 2022, USA [14] | Prospective cohort study | 377 ambulatory patients with chronic systolic heart failure (100 Black, 277 White), across 21 ventricular assist device (VAD) centers in 16 U.S. states, 2015–2016, from the REVIVAL study (Registry Evaluation of Vital Information for VADs in Ambulatory Life) | To assess racial disparities in the utilization of ventricular assist devices and heart transplants, accounting for clinical, social, and preference-based factors | Black patients had a 55% lower likelihood of receiving ventricular assist devices or transplant (adjusted hazard ratio 0.45). Disparities persisted after adjusting for heart failure severity, patient preferences, quality of life, and social determinants of health. Black patients also had higher mortality (18% vs. 13%). | Structural racism embedded in advanced heart failure care contributes to disparities in life-saving treatments through systemic bias and unequal institutional practices, not patient preference or clinical need, reinforcing inequities in survival outcomes. |
| Chambers et al., 2019, USA [82] | Retrospective cohort study | 47,771 non-Hispanic Black women in California (2011–2012 birth cohort) | To assess whether residential racial and economic segregation, measured by the Index of Concentration at the Extremes, is associated with preterm birth and infant mortality among Black women | Black women in the least privileged zip codes had 25–32% higher odds of preterm birth and 54–68% higher odds of infant mortality compared to those in the most privileged areas. The Index of Concentration at the Extremes remained a strong predictor after adjustment for confounders. | Structural racism, explicitly quantified through racial and economic segregation, produces disparities in birth outcomes among Black women by shaping neighborhood-level deprivation, differential healthcare access, and chronic stress exposures at the community level. |
| Chan et al., 2024, USA [44] | Population-based cohort study | 288,787 pregnant individuals in Massachusetts, United States (1995–2015), using data from the Massachusetts Birth Registry | To examine associations between historical redlining and pregnancy-related chronic conditions and birth outcomes | Higher redlining grades (B–D) were associated with increased risks of pregestational diabetes (relative risk 1.7 for Grade D), chronic hypertension (relative risk 1.5 for Grade D), gestational diabetes (relative risk 1.5), hypertensive disorders, small-for-gestational-age infants, and low birth weight. Associations remained significant after accounting for current segregation. | Structural racism embedded in historical redlining policies continues to produce disparities in maternal health and birth outcomes through pathways of environmental inequity, chronic stress, neighborhood deprivation, and reduced access to high-quality healthcare. |
| Chegwin et al., 2023, USA [83] | Ecological cross-sectional study | 75,461 Black and 278,372 White women in 430 New Jersey municipalities (2012–2016), using linked birth records and police use of force data from The Force Report | To examine associations between racialized police use of force and birth outcomes, disaggregated by race | Racialized police use of force, measured as disproportionate use against Black individuals, was significantly associated with higher odds of low birth weight (β = 0.06) and preterm birth (β = 0.06) among Black women. No associations were observed for White women or with overall police use of force rates. | Structural racism, operationalized through racialized policing practices, contributes to adverse maternal and infant health outcomes among Black women by perpetuating chronic stress, community trauma, and systemic exposure to discriminatory policing, independent of individual socioeconomic status. |
| Chen et al., 2022, USA [84] | Ecological cross-sectional study | 20,782 youth (0–18 years) in Los Angeles County (2016–2019) who received Mobile Crisis Response services, based on administrative and neighborhood-level data. | To examine associations between neighborhood opportunity, inequality, racial composition, and psychiatric emergency response frequency | Black and multiracial youth were overrepresented in Mobile Crisis Response cases. Higher Black youth population density and education opportunity inequality predicted increased crisis response incidents. Lower neighborhood opportunity and higher inequality were associated with greater psychiatric emergencies. | Structural racism, operating through unequal neighborhood resources, racial segregation, and institutional biases, contributes to disparities in youth mental health outcomes and patterns of emergency service use among racially and ethnically minoritized youth populations. |
| Collin et al., 2021, USA [11] | Population-based retrospective cohort study | 8,523 non-Hispanic Black and White women with invasive breast cancer in metropolitan Atlanta, United States (2010–2014), using Georgia Cancer Registry data | To examine associations between neighborhood-level redlining and lending bias and breast cancer mortality | Redlining was associated with a 1.6 times higher breast cancer mortality risk (HR = 1.58). Lending bias was associated with a 14% lower mortality risk (HR = 0.86), possibly reflecting benefits in affluent areas. Eighty percent of non-Hispanic Black women versus 20% of non-Hispanic White women lived in redlined neighbourhoods. | Structural racism, mediated through both historical redlining and ongoing lending bias, produces disparities in breast cancer mortality by perpetuating spatial segregation, differential healthcare access, and unequal distribution of healthcare and community resources. |
| Daoud et al., 2022, Israel [75] | Qualitative study | 10 hospital directors, 40 nurses/midwives, and 26 Jewish and Palestinian-Arab birthing women; public hospitals in northern, central, and southern Israel (2019–2021) | To examine the mechanisms of racial maternal separation in maternity wards and their role in creating inequitable maternal care | Racial maternal separation disproportionately targeted Palestinian-Arab Muslim women. Separation was institutionalized under the guise of cultural sensitivity and commodified healthcare, rooted in structural segregation and economic incentives rather than patient preference. Hospital administrators were aware but rarely intervened. | Structural racism within Israeli maternity care operates through racial maternal separation practices, which institutionalize discriminatory treatment under culturally framed policies, reinforcing ethnic inequities in maternal care and exacerbating structural segregation within the healthcare system. |
| Davis et al., 2023, USA [60] | Ecological cross-sectional study | 4,270,207 U.S.-born mothers with singleton live births across 3,135 U.S. counties (2011–2015), using NCHS vital statistics and 2012 Census of Governments | To assess whether county-level reliance on law enforcement fees and fines for municipal revenue is associated with adverse birth outcomes | Counties in the highest quartile of fee and fine reliance had significantly higher odds of preterm birth (OR = 1.075) and low birth weight (OR = 1.066). Associations remained significant after adjusting for race, income, and residential segregation. | Structural racism, operationalized through racially punitive municipal fiscal policies such as excessive reliance on fines and fees, contributes to disparities in maternal and infant health outcomes by imposing economic stress and systemic disadvantage disproportionately on racially minoritized communities. |
| English et al., 2024, USA [29] | Cross-sectional study | Cross-sectional study (2021); 5,064 Black and White heterosexual and LGBQ adults; New Jersey, Minnesota, Mississippi (USA) | To examine how racist and heterosexist criminal legal policies influence police stops, discrimination, and suicidality across intersecting identities | Racist and heterosexist policies decreased police stops for White heterosexuals but increased stops for Black LGBQ individuals. Police stops and discrimination mediated suicidality among Black LGBQ participants. Policies were protective for White heterosexuals but harmful for Black LGBQ individuals. | Structural oppression embedded in state-level criminal legal policies—including racism and heterosexism—contributes to suicide-related health disparities by increasing exposure to policing and discrimination among Black LGBQ populations, exacerbating psychosocial stress and health risks through intersectional pathways. |
| Francis et al., 2023, USA [76] | Qualitative study | 20 Black women aged 18–44 with a history of hypertensive disorders of pregnancy, Greater New Haven, Connecticut, USA, 2020 | To explore how interconnected domains of racism affect Black women’s lived pregnancy experiences in the context of hypertensive disorders of pregnancy | Five overlapping domains of structural racism were identified: medical discrimination, underfunded infrastructure, housing inequities, environmental pollution, and policing and surveillance. Four intersecting pathways were described that worsen hypertensive disorders of pregnancy and related health behaviors. | Structural racism operates across multiple interconnected domains—including healthcare, housing, environment, and policing—compounding stress, limiting care access, and exacerbating maternal health disparities among Black women with hypertensive disorders of pregnancy. |
| Gadela et al., 2022, USA [27] | Descriptive epidemiological study | 2,731 children aged 1–14 years who died from asthma, United States, from 1999 to 2018, based on CDC WONDER Multiple Cause of Death Files | To examine racial inequalities and trends in pediatric asthma mortality and to generate hypotheses for addressing these inequities through clinical and public health interventions | Black children had 6.43–7.54 times higher mortality rates from pediatric asthma compared to White children. Mortality rates were highest in urban areas for Black children and in rural areas for White boys. Racial inequities persisted over time. | Structural racism, operating through under-resourced communities, economic marginalization, and residential segregation, contributes to persistent disparities in pediatric asthma mortality by shaping differential environmental exposures, healthcare access, and chronic stress in Black communities. |
| Gao et al., 2024, USA [10] | Cross-sectional study | 1,554,837 live singleton births (≥20 weeks’ gestation) in California, USA (2005–2018) | To examine associations between historical redlining, contemporary gentrification, and severe maternal morbidity in California | Living in historically redlined neighborhoods undergoing gentrification or displacement was associated with higher odds of severe maternal morbidity. Black, Hispanic, and American Indian or Alaskan Native birthing individuals were disproportionately affected. | Structural racism, through the combined effects of historical redlining and contemporary gentrification, perpetuates maternal health disparities by displacing marginalized communities, increasing housing and social stressors, and compounding barriers to high-quality maternal care. |
| Guglielminotti et al., 2024, USA [54] | Population-based retrospective cohort study | Non-Hispanic Black women (n = 1,081,078) and non-Hispanic White women (n = 3,723,410) from the 2017–2018 US birth certificates | To examine associations between state-level structural racism indicators and severe adverse maternal outcomes during childbirth | Higher Black-to-White inequity ratios in unemployment and incarceration were significantly associated with increased odds of severe adverse maternal outcomes among Black women: 35% increase per one-unit increase in unemployment ratio and 6% increase per one-unit increase in incarceration ratio. White women also showed weaker associations. No significant associations were found for education inequity ratios. | Structural racism, quantified through state-level Black-to-White inequity ratios in unemployment and incarceration, contributes to disparities in severe maternal outcomes among Black women by embedding systemic disadvantage and cumulative stress, independent of individual socioeconomic characteristics. |
| Hailu et al., 2024, USA [23] | Population-based cross-sectional study | 10,200,692 live births in California from 1997 to 2018 | To examine associations between county-level jail incarceration inequity (Black vs. White) and severe maternal morbidity risk | Black and Hispanic birthing individuals in high-inequity counties had higher odds of severe maternal morbidity (Black: OR 1.14–1.20; Hispanic: OR 1.20–1.24). White birthing individuals showed weaker associations. | Structural racism, operating through racial inequities in the criminal legal system and associated mass incarceration, contributes to disparities in maternal health outcomes by increasing exposure to systemic stressors and undermining healthcare access for racially and ethnically minoritized populations. |
| Harville et al., 2022, USA [68] | Multilevel cross-sectional study | 2,950,965 births across 5,924 counties in 45 U.S. states (2015–2016), including non-Hispanic White, non-Hispanic Black, and Hispanic mothers | To examine associations between county-level eviction rates and adverse birth outcomes, with a focus on racial disparities | Living in counties with the highest eviction rates was associated with 12–13% increased odds of low birth weight and preterm birth. Non-Hispanic Black women were more likely to reside in high-eviction counties and experienced stronger adverse effects. | Structural racism, operationalized through housing instability and disproportionate exposure to eviction, contributes to racial disparities in adverse birth outcomes by increasing economic stress and undermining maternal health, particularly for Black women. |
| Havens et al., 2011, USA [77] | Qualitative process evaluation | 28 staff, supervisors, and Board of Health members from a Southern County Public Health Department in North Carolina | To explore factors influencing participation in an antiracism initiative and perceptions of institutional racism | Participation in the Dismantling Racism initiative was shaped by personal experiences with racism, perceived relevance to job roles, and expectations of impact. The initiative increased awareness of racism but also led to workplace tensions among some staff. | Structural racism is embedded in public health systems. Structured antiracism training can increase awareness and shift organizational perspectives, but addressing institutional racism requires systemic change and sustained commitment to equity. |
| OjiNjideka Hemphill  et al., 2023, USA [19] | Qualitative study | 11 young Black pregnant women (ages 18–24) in Chicago, Illinois | To explore maternal health and pregnancy experiences of young Black women through an intersectional lens | Participants reported obstetric racism, including dismissal of concerns, lack of informed consent, and medical mistrust. Participants resisted these experiences through self-advocacy and reliance on trusted providers. | Structural racism, operating through implicit bias and systemic inequities in maternity care, undermines healthcare quality for young Black women and contributes to adverse maternal health experiences and outcomes. |
| Henderson and Quenby, 2025, UK [85] | Observational cohort study | 17,701 women with singleton pregnancies from the UK Millennium Cohort Study | To examine how socioeconomic position mediates ethnic disparities in preterm birth and small-for-gestational-age outcomes | Black African, Black Caribbean, and Indian women had the highest preterm birth rates; Indian, Pakistani, and Bangladeshi women had the highest small-for-gestational-age rates. Latent socioeconomic position explained up to 60% of preterm birth and 53% of small-for-gestational-age disparities. Traditional socioeconomic measures underestimated disparities. | Structural racism operates through socioeconomic stratification and cumulative disadvantage, driving ethnic disparities in perinatal outcomes by shaping differential access to resources, neighborhood exposures, and healthcare quality. |
| Hernandez et al., 2024, USA [86] | Prospective cohort study | 342 breast cancer patients (54.4% Hispanic, 17.3% Black, 15.8% White) from a National Cancer Institute cancer center and safety-net hospital in Miami, Florida | To assess how perceived discrimination influences mammography use and breast cancer stage at diagnosis | Higher perceived discrimination was associated with lower screening mammography rates (adjusted odds ratio 0.956) and increased odds of late-stage breast cancer diagnosis (adjusted odds ratio 1.062). Discrimination was strongly associated with mistrust of healthcare providers. | Structural racism, mediated through interpersonal discrimination, provider mistrust, and neighborhood disadvantage, contributes to disparities in breast cancer outcomes among racially and ethnically minoritized women by undermining preventive care and early detection. |
| Hollenbach et al., 2021, USA [45] | Retrospective cohort study | 64,804 live births from 15 ZIP codes in the Finger Lakes Region, New York (2005–2018) | To evaluate associations between historical redlining and current obstetric and neonatal health disparities | Preterm birth rates were significantly higher in historically redlined areas (12.38%) compared to “Best/Still Desirable” areas (7.55%). Associations remained after adjusting for race and socioeconomic status. Additional disparities were observed in maternal depression, substance use disorder, breastfeeding rates, and NICU admissions. | Structural racism, through the legacy of redlining, contributes to persistent disparities in maternal and neonatal health by shaping neighborhood deprivation, psychosocial stress, and access to perinatal care in historically disinvested communities. |
| Homan and Brown, 2022, USA [33] | Cross-sectional study | 11,900 adults aged 51+ years (2,957 Black and 8,943 White) across 35 U.S. states | To examine associations between racialized felony disenfranchisement and health outcomes among older Black and White adults | Higher levels of racialized felony disenfranchisement were significantly associated with increased depressive symptoms, functional limitations, and difficulties with instrumental and basic activities of daily living among Black adults. No significant associations were found among White adults. | Structural racism, operationalized through racialized felony disenfranchisement and political exclusion, contributes to disproportionate mental and physical health burdens among Black adults by reinforcing systemic marginalization and limiting social and civic participation. |
| Hung et al., 2022, USA [55] | Retrospective cohort study | 166,791 women who gave birth in South Carolina between 2018 and 2021 | To examine racial and ethnic disparities in severe maternal morbidity before and during the COVID-19 pandemic, and whether disparities varied by Black residential segregation | Black and Hispanic women living in highly segregated Black communities had higher odds of severe maternal morbidity compared to their counterparts in less segregated areas. During the COVID-19 pandemic, Black–White disparities persisted, and Hispanic–White disparities worsened. Residential segregation exacerbated disparities in severe maternal morbidity. | Structural racism, particularly through residential segregation, contributes to disparities in severe maternal morbidity among Black and Hispanic women by embedding differential access to care, economic hardship, and cumulative disadvantage, effects that were further exacerbated during the COVID-19 pandemic. |
| Hunte et al., 2022, USA [78] | Qualitative study | Seven Black female nurses and community health workers from the Healthy Birth Initiatives program in Portland, Oregon. | To explore the experiences of Black perinatal care providers delivering culturally specific care to Black families | Shared racial identity between providers and clients fostered trust and improved engagement. Providers reported navigating racism-related stress and systemic biases while delivering care. Black providers experienced a dual burden of client care and systemic oppression. Underfunding of culturally specific programs limited their reach and sustainability. | Structural racism operates both through systemic underfunding of culturally specific community health programs and through the undervaluation and overburdening of Black healthcare workers, constraining the sustainability and reach of equity-focused perinatal care for Black families. |
| Igbinosa et al., 2023, USA [87] | Population-based cohort study | 3,863,594 pregnant individuals, including singleton births, in California (2011–2020) | To examine racial and ethnic disparities in antepartum anemia and its contribution to severe maternal morbidity | Black patients had the highest anemia prevalence (21.5% in 2020), which had doubled since 2011. Anemia contributed to 20.9% of severe maternal morbidity cases among Black patients. Persistent racial disparities in anemia and severe maternal morbidity remained after adjustment for social determinants and clinical factors. Anemia explained part of the disparities for Black, Hispanic, and multiracial patients. | Structural racism operates through race-based clinical practices, unequal screening, and inequitable treatment pathways, contributing to racial disparities in maternal health outcomes by systematically failing to provide equitable prevention and management of antepartum anemia among racially minoritized groups. |
| Jadow et al., 2023, USA [12] | Ecological cross-sectional study | 2,117 census tracts in New York City (2014–2018) | To assess associations between historical redlining, social determinants of health, and stroke prevalence across neighborhoods | Historical redlining was independently associated with higher stroke prevalence, even after adjustment for social determinants of health and cardiovascular risk factors. Redlining ranked 7th in variable importance, following income, education, and healthcare access. Poverty and low educational attainment were also significantly associated with stroke disparities. | Structural racism, through the enduring impact of discriminatory housing policy (redlining), continues to produce disparities in stroke prevalence by shaping neighborhood-level socioeconomic deprivation, environmental exposures, and cumulative disadvantage decades after the original redlining policies. |
| Jahn et al., 2020, USA [22] | Ecological cross-sectional study | 41,911,094 births to non-Hispanic Black and White women in the United States (1999–2015) | To examine associations between county-level jail incarceration rates and preterm birth | Higher county-level jail incarceration rates were associated with greater odds of preterm birth for both Black and White women. The effect was stronger in high-incarceration areas. Adjusting for Black–White incarceration disparities reduced racial inequities in preterm birth. | Structural racism within the criminal justice system, operationalized through disproportionate incarceration of Black individuals, contributes to adverse perinatal outcomes by generating chronic community-level stress, disrupting social networks, and widening racial health disparities across the broader community. |
| Jahn et al., 2021, USA [24] | Ecological time-series study | 7,709,300 live births across 520 Core-Based Statistical Areas (CBSAs), stratified by racial and ethnic group (2013–2015) | To examine whether fatal police violence is associated with pregnancy loss, and whether effects vary by race and racial concordance of the fatality | Each fatal police event during early gestation was associated with a 0.14% decrease in live births overall and a 0.29% decrease among Black women. No significant effects were observed for White women. Effects were strongest in Black and Hispanic populations and in areas with high police violence rates. | Structural racism, through racialized police violence, produces disparities in reproductive outcomes by generating chronic stress, collective trauma, and racialized hypervigilance that disproportionately harm Black and Hispanic women's reproductive health and contribute to intergenerational health inequities. |
| Jahn et al., 2023, USA [7] | Multilevel cross-sectional study | 9,102 births in New Orleans, Louisiana (2018–2019), focusing on Black and White birthing people | To measure neighborhood exposure to proactive policing as a manifestation of structural racism and its association with preterm birth | Black birthing people in neighborhoods with the highest rates of proactive policing had 1.41 times higher prevalence of preterm birth compared to those in neighborhoods with the lowest rates. No significant association was found for White birthing people. | Structural racism, operationalized through proactive policing practices disproportionately targeting Black communities, contributes to disparities in preterm birth by inducing chronic stress, racialized hypervigilance, and systemic barriers to safe and supportive environments for Black birthing individuals. |
| James and Horne, 2024, USA [88] | Cross-sectional study | 780 Black American adults (nationwide, USA) | To examine associations between structural barriers to healthcare access, internalized racism, and attitudes toward seeking healthcare across mental, medical, dental, and vision care | Structural barriers to healthcare access, including lack of insurance, transportation issues, and discrimination, were associated with internalized racism, which in turn was linked to negative attitudes toward seeking healthcare. Barriers to dental care showed unique patterns not fully explained by internalized racism. | Structural racism operates through systemic barriers to healthcare access, which foster internalized racism and contribute to negative health-seeking attitudes among Black Americans, perpetuating inequities in healthcare utilization and outcomes. |
| Janevic et al., 2025, USA [32] | Prospective cohort study | 373 Asian, Black, and Hispanic postpartum individuals in New York City and Philadelphia | To examine associations between gendered racial microaggressions and postpartum blood pressure | 37.5% of participants experienced at least one gendered racial microaggression during obstetric care. Gendered racial microaggressions were associated with higher postpartum blood pressure, particularly after 12 days postpartum. Strongest associations were observed among individuals with hypertensive disorders of pregnancy and high Structural Racism Effect Index exposure. | Structural racism, operating through both interpersonal (gendered racial microaggressions) and neighborhood-level pathways, contributes to disparities in maternal cardiovascular outcomes by inducing chronic stress and compounding the impact of existing health inequities among racially and ethnically minoritized postpartum individuals. |
| Jeffers et al., 2023, USA [69] | Multilevel cross-sectional study | 234,917 Black birthing people in the U.S. for racialized economic segregation analysis; 220,624 for Black–White incarceration inequality analysis | To investigate associations between racialized economic segregation and incarceration inequality and severe maternal morbidity among Black birthing people | Racialized economic segregation (measured by Index of Concentration at the Extremes for race and income) was associated with increased odds of severe maternal morbidity. Black–White incarceration inequality was not significantly associated with severe maternal morbidity. | Structural racism, operating through racialized economic segregation, increases maternal health disparities among Black birthing people by embedding systemic deprivation and cumulative disadvantage in segregated neighborhoods, which exacerbates risks for adverse maternal outcomes. |
| Karvonen et al., 2022, USA [47] | Observational cohort study | 13,321 Black birthing people with preterm infants (22–36 weeks) in California (2011–2017) | To examine whether structural racism, measured by the Index of Concentration at the Extremes, affects postnatal outcomes for Black preterm infants | Infants in the least privileged Index of Concentration at the Extremes tertiles had higher risks of ≥2 acute care visits (relative risk 1.27), rehospitalization (relative risk 1.10), and post-discharge mortality (relative risk 1.92). Disparities persisted after adjustment for clinical and social factors. | Structural racism, quantified via racial and economic segregation, contributes to disparities in postnatal healthcare outcomes among Black preterm infants by shaping neighborhood-level disadvantage, healthcare quality, and differential exposure to structural stressors. |
| Karvonen et al., 2025, USA [46] | Retrospective cohort study | 4,355 children, adolescents, and young adults (<40 years) diagnosed with cancer in Seattle and Tacoma (2000–2019) | To assess whether historical redlining is associated with survival outcomes in pediatric and adolescent/young adult cancer patients | Patients from historically redlined neighborhoods had worse five-year survival (85.1% vs. 90.3%) and ten-year survival (81.1% vs. 88.1%). Young adults (aged 18–39) in redlined areas had a 34% higher mortality risk (hazard ratio 1.34). No significant survival differences were observed among pediatric patients (<18 years), possibly due to stronger Medicaid protections. | Structural racism, through the enduring effects of historical redlining, continues to shape disparities in cancer survival outcomes among racially and economically marginalized youth by influencing access to high-quality care, environmental exposures, and cumulative social disadvantage. |
| Khanijahani and Tomassoni, 2022, USA [61] | Ecological cross-sectional study | 73,056 census tracts across 3,142 U.S. counties | To examine associations between county-level COVID-19 mortality and residential racial and socioeconomic segregation | Counties with higher proportions of residents in concentrated disadvantage or Black-concentrated tracts had 14% and 11% higher COVID-19 death rates, respectively. Joint segregation further amplified mortality disparities. | Structural racism, operating through residential racial and socioeconomic segregation, exacerbated COVID-19 mortality disparities by concentrating structural disadvantage, limiting healthcare access, and increasing exposure to risk among marginalized communities during the pandemic. |
| Lee et al., 2024, USA [89] | Cross-sectional study | 9,907 live births across 17 U.S. states (2016–2020) | To examine associations between perceived racism and hypertensive disorders of pregnancy, and whether adjustment for racism reduces racial and ethnic disparities in hypertensive disorders of pregnancy diagnoses | Non-Hispanic Black individuals had the highest hypertensive disorders of pregnancy rates (21.8%). Overall, 18% of participants were diagnosed with hypertensive disorders of pregnancy; 76.4% reported experiences of racism and/or discrimination. While perceived racism did not independently increase hypertensive disorders of pregnancy risk, adjusting for racism reduced Black–White disparities in hypertensive disorders of pregnancy. | Structural racism influences maternal health outcomes through cumulative stress, constrained healthcare access, and negative healthcare experiences, contributing to racial disparities in hypertensive disorders of pregnancy. Racism and discrimination embedded in healthcare interactions shape health inequities beyond biological differences. |
| Li et al., 2024, USA [48] | Retrospective cohort study | 162,587 non-Hispanic Black and White adult candidates for first-time live donor kidney transplantation across the U.S. | To examine whether residential and transplant center neighborhood segregation is associated with access to live donor kidney transplantation | Black candidates in high-segregation residential neighborhoods had 10% lower access to live donor kidney transplantation than those in low-segregation areas. Both Black and White candidates at high-segregation transplant centers had lower access to live donor kidney transplantation. Black candidates in predominantly minority neighborhoods had 65% lower likelihood of live donor kidney transplantation than White candidates in predominantly White neighborhoods. | Structural racism, operating through both residential and institutional segregation, limits access to life-saving kidney transplantation for Black patients by shaping healthcare infrastructure, care pathways, and systemic inequities in referral, evaluation, and donor matching processes. |
| Lubarsky et al., 2024, USA [16] | Retrospective cohort study | 5,173 women diagnosed with stage I–IV breast cancer treated at the University of Miami and Jackson Health System (2005–2017) | To assess whether structural racism, measured by the Index of Concentration at the Extremes, affects receipt of NCCN guideline-concordant treatment | Non-Hispanic Black women were 42–47% less likely to receive NCCN-concordant treatment than Non-Hispanic White women across all Index of Concentration at the Extremes models. Hispanic patients in highly segregated neighborhoods also faced care disparities. Disparities persisted independent of residential or economic segregation. | Structural racism, extending beyond neighborhood segregation, contributes to treatment disparities through systemic barriers including provider bias, differential healthcare access, and medical mistrust, undermining the quality of cancer care for racially and ethnically minoritized women. |
| Ly et al., 2023, USA [49] | Retrospective cohort study | 1,868,036 Medicare beneficiaries (Black and White adults aged 65–99 years) undergoing eight common surgical procedures | To assess racial and sex inequities in surgical mortality across elective and non-elective surgeries | Black men had the highest postoperative mortality, particularly after elective surgeries (50% higher than White men). Disparities persisted at 7, 14, 30, and 60 days post-surgery. Differential surgeon distribution explained approximately 30% of the disparity. Additional contributors included hospital quality and neighborhood disadvantage. | Structural racism embedded within healthcare systems perpetuates racial disparities in surgical outcomes through inequitable access to high-quality surgeons and hospitals, cumulative impacts of neighborhood disadvantage, and systemic healthcare biases that disproportionately harm Black men. |
| Machado et al., 2021, Brazil [67] | Longitudinal cohort study | 10,130 Brazilian civil servants aged 35–74 years who were non-obese at baseline | To examine associations between race/skin color, perceived racial discrimination, and obesity incidence over a four-year follow-up, stratified by education level | Black individuals with high education had a 2.22-fold higher risk of developing obesity compared to White peers. Among Black individuals with low education, perceived racial discrimination was associated with a 64% increased obesity risk (odds ratio 1.64). No significant associations were observed for Brown individuals. | Structural racism, operating through both institutional and interpersonal pathways, intersects with educational status to shape obesity risk by inducing chronic stress, limiting access to protective resources, and exacerbating health inequities among Black individuals. |
| Mahabir et al., 2021, Canada [79] | Qualitative study | 41 racialized healthcare users, 23 non-racialized users, and 11 providers in Toronto/GTA | To examine how healthcare policies and practices affect racialized populations in Toronto’s healthcare system | Five clusters of mistreatment were identified: racial/class discrimination, dehumanization, negligent communication, professional misconduct, and unequal access. Two overarching themes emerged: being “viewed as inferior” and receiving “unequal medical care”. Racialized users consistently reported systemic racism as a central barrier to equitable care. | Structural racism is embedded across multiple healthcare system levels, with institutional, meso-, and macro-level structures perpetuating inequities in care for racialized users through power imbalances, exclusionary biomedical models, and neoliberal healthcare practices. |
| Maldonado et al., 2022, USA [30] | Cross-sectional study | 433 non-Hispanic Black adults from the Wave 4 cohort (2013–2017) of the Healthy Aging in Neighborhoods of Diversity across the Life Span (HANDLS) study in Baltimore, Maryland | To examine associations between racial discrimination and intimate partner violence perpetration via mental health symptoms, with moderation by poverty or gender | Racial discrimination led to worsened mental health symptoms (depression, anxiety, PTSD), which increased intimate partner violence perpetration. Effects were stronger among Black women and those living in poverty. Overall, 71% of participants reported intimate partner violence, and 70% reported psychological aggression. | Structural racism, through racial discrimination and its impact on mental health, contributes to the elevated risk of intimate partner violence, particularly among multiply marginalized Black individuals, by exacerbating psychosocial stress and undermining community resilience. |
| Matoba et al., 2019, USA [90] | Retrospective cross-sectional study | 33,586 African American mothers in Chicago, Illinois, from 1989 to 1991 | To examine associations between mortgage discrimination (redlining) and preterm birth, and its relation to racial residential segregation | Preterm birth rates were higher in redlined areas (18.5%) compared to non-redlined areas (17.1%). Adjusted odds ratio for preterm birth in redlined areas was 1.12 (95% confidence interval: 1.04–1.20). Strongest associations were observed in highly segregated African American neighborhoods. | Structural racism in housing, through redlining and its interaction with racial residential segregation, contributes to racial disparities in preterm birth by reinforcing spatial inequities, economic disadvantage, and psychosocial stress within marginalized communities. |
| Matthews et al., 2021, USA [18] | Qualitative study | 10 Black women stakeholders in perinatal mental health (clinicians, researchers, activists), United States (national) | To identify strategies to address structural racism and inequities in maternal mental health care for Black birthing people | Structural racism resulted in underdiagnosis and undertreatment of mental health conditions among Black women. Stakeholders identified five strategies for equity: educating and training providers, expanding the Black mental health workforce, supporting Black-led organizations, integrating traditional healing practices, and promoting shared decision-making and integrated care. Racism and gender oppression compounded mental health risks. | Structural racism perpetuates maternal mental health disparities among Black women by embedding biased diagnostic practices, underinvestment in culturally concordant care, and systemic exclusion of Black-led and community-based services, reinforcing both clinical and structural inequities. |
| McGrath et al., 2023, USA [72] | Retrospective cohort study | 8,269 pediatric patients with central catheters treated at Seattle Children’s Hospital between 2012 and 2022 | To examine disparities in central line–associated bloodstream infection rates among minoritized racial, ethnic, and language groups, and evaluate the effectiveness of equity-focused interventions | Black patients had an unadjusted central line–associated bloodstream infection rate of 2.8 per 1000 catheter days; non-English speakers had a rate of 2.1 versus 1.5 overall. After adjustment, Black patients had an adjusted hazard ratio of 1.8, and non-English speakers had an adjusted hazard ratio of 1.6. Implementation of targeted equity-focused interventions significantly reduced these disparities. | Structural racism, embedded in clinical infection prevention practices, contributes to disparities in healthcare-associated infections among racially and linguistically minoritized children by perpetuating inequities in care processes, communication, and clinical vigilance. Equity-focused interventions addressing these systemic biases can reduce disparities and promote safer pediatric care. |
| Mendez et al., 2011, USA [56] | Cross-sectional study | 4,880 pregnant women in Philadelphia, United States | To develop a residential redlining index using Home Mortgage Disclosure Act data and assess its association with racial segregation and pregnancy-related health outcomes | Black applicants were twice as likely to be denied mortgage loans as White applicants (odds ratio 2.00–2.26). 77.7% of pregnant women lived in redlined neighborhoods, disproportionately affecting Black women. Redlining strongly correlated with segregation indices (dissimilarity, isolation). | Structural racism, operationalized via mortgage discrimination, produces neighborhood-level structural disadvantage and contributes to racial disparities in perinatal health outcomes by constraining housing opportunities, concentrating poverty, and generating cumulative social stress for Black women. |
| Miller-Kleinhenz et al., 2024, USA [50] | Population-based cohort study | 1,764 non-Hispanic Black and White women diagnosed with breast cancer in Georgia between 2010 and 2017 | To examine associations between historical redlining, contemporary mortgage discrimination, persistent mortgage discrimination, and breast cancer outcomes | Historical redlining areas (score ≥2.5) were associated with 62% higher odds of estrogen receptor-negative breast cancer among Black women. Historical redlining was linked to 97% higher odds of late-stage breast cancer among White women. Persistent mortgage discrimination was associated with a 60% increased breast cancer mortality, especially among White women. Black women exhibited elevated mortality risk regardless of persistent mortgage discrimination. | Structural racism, through both historical and ongoing housing discrimination, contributes to disparities in breast cancer outcomes across racial groups by shaping neighborhood-level disadvantage, healthcare access, environmental exposures, and cumulative social stress, thereby perpetuating inequities in cancer progression and survival. |
| Mohottige et al., 2023, USA [91] | Cross-sectional ecological study | 150 residential census block groups in Durham County, North Carolina | To examine associations between structural racism indicators and neighborhood prevalence of chronic kidney disease, diabetes, and hypertension | Greater structural racism, measured via indicators such as the Index of Concentration at the Extremes for race and income, proportion of White residents, Area Deprivation Index, and discrete indicators (crime, evictions, education), was linked to higher prevalence of chronic kidney disease, diabetes, and hypertension. | Structural racism, manifesting through residential segregation, economic disparities, and policy-driven inequities, contributes to the elevated prevalence of chronic diseases by shaping social determinants of health, limiting healthcare access, and fostering cumulative disadvantage in marginalized communities. |
| Nardone et al., 2020, USA [57] | Retrospective cohort study | 1,627,214 births (2006–2015) in Los Angeles, Oakland, and San Francisco within 1930s HOLC-mapped areas | To examine associations between historical redlining and adverse birth outcomes in California | Higher odds of preterm birth and small-for-gestational-age outcomes were observed in grade C versus grade B neighborhoods. Lower odds of preterm birth, low birth weight, and small-for-gestational-age outcomes were observed in grade D versus grade C neighborhoods, potentially reflecting gentrification effects. Disparities varied by metropolitan area and by maternal race. | Structural racism, through historical redlining policies, continues to shape adverse birth outcomes by generating lasting neighborhood deprivation, disinvestment, and cumulative psychosocial stress among racially minoritized populations, perpetuating spatial health inequities across generations. |
| Nguyen et al., 2022, USA [92] | Cross-sectional study | 242,274 nonelderly Medicaid managed care enrollees across 37 U.S. states | To examine racial and ethnic disparities in patient experience metrics across Medicaid managed care plans | Black, Hispanic/Latino, and Asian/Pacific Islander enrollees reported significantly worse patient care experiences than White enrollees (disparities ranged from 1.5 to 17.4 percentage points). Disparities were primarily within plans rather than between plans. Plans with higher proportions of minoritized enrollees exhibited smaller disparities. | Structural racism embedded within Medicaid managed care systems contributes to unequal healthcare experiences by producing within-plan inequities in patient-centered care for racial and ethnic minority groups, perpetuating systemic barriers to high-quality healthcare despite nominal insurance coverage. |
| Nordyke et al., 2023, USA [80] | Qualitative study | 25 adults (17 women, 8 men) from Somali, Hmong, Black/African American, Hispanic/Latino/a, and First Nations/Native American/Indigenous communities in Wisconsin | To explore the impact of racism on healthcare experiences and well-being for communities of color by examining lived experiences, barriers, and systemic factors contributing to healthcare disparities | Systemic racism negatively impacted healthcare experiences and well-being. Participants identified three key factors—backgrounds and values, resources, and prejudices—that required constant navigation and vigilance, diminishing healthcare access. Cultural mismatches in healthcare settings further exacerbated disparities. | Structural racism, operating across institutional, interpersonal, and internalized domains, entrenches systemic barriers to equitable healthcare for marginalized communities by shaping clinical interactions, diminishing cultural concordance, and fostering mistrust, thereby reinforcing health inequities and limiting healthcare engagement. |
| Poisson et al., 2024, USA [21] | Retrospective cohort study | 38 pediatric patients with aquaporin-4 antibody–positive neuromyelitis optica spectrum disorder (AQP4+ NMOSD) from 3 U.S. tertiary centers (2009–2021) | To evaluate racial, ethnic, and socioeconomic disparities in pediatric AQP4+ neuromyelitis optica spectrum disorder outcomes | Black/African American children had significantly higher disability (mean Expanded Disability Status Scale: 2.46 vs. 0.33; p=0.003), more hospitalizations (2.37 additional admissions; p=0.002), and longer inpatient stays (28.4 additional days; p=0.002) compared to White children. Public insurance was associated with higher relapse rates (p=0.046). Neighborhood deprivation (poverty, low income, vacant housing) strongly correlated with worse disability (p<0.05). | Structural racism and intersecting social determinants—including public insurance status, neighborhood poverty, and systemic discrimination—drive disparities in disability, healthcare access, and health outcomes in pediatric neuromyelitis optica spectrum disorder by shaping exposure to cumulative disadvantage across healthcare and community contexts. |
| Poulson et al., 2021, USA [51] | Retrospective cohort study | 296,597 Black and White colorectal cancer patients (2005–2015), US Surveillance, Epidemiology, and End Results data | To examine the influence of racial residential segregation on disparities in colorectal cancer diagnosis, treatment, and survival | Black patients living in highly segregated areas had a 41% higher risk of advanced-stage diagnosis and a 43% higher risk of cancer-specific mortality. Racial disparities in surgical resection disappeared in low-segregation areas. | Residential segregation, rooted in historical and systemic racism, is a key determinant of colorectal cancer disparities by concentrating structural disadvantage, constraining access to timely screening and treatment, and perpetuating inequities in cancer outcomes for Black patients. |
| Quinn et al., 2024, USA [26] | Cross-sectional survey | 538 Black/African American adults in Chicago | To examine how neighborhood violence, police violence, and racism relate to COVID-19 vaccination, mediated by trust and mental health | Neighborhood violence and racism increased medical mistrust and depressive symptoms. Medical mistrust reduced trust in COVID-19 information from physicians, lowering vaccination rates. Police violence was associated with higher depressive symptoms. | Structural racism, operating through community-level violence and interpersonal racism, erodes trust in healthcare, exacerbates mental health burdens, and contributes to lower vaccine uptake among Black communities by undermining confidence in health authorities and perpetuating systemic mistrust. |
| Ramos et al., 2024, USA [8] | Ecological cross-sectional study | 8,042,672 births and 48,352 infant deaths across 1,181 U.S. counties (2016–2019) | To examine associations between a Systemic Racism Index and non-Hispanic Black versus non-Hispanic White infant mortality disparities | Each one standard deviation increase in the Systemic Racism Index was associated with a 10.4% increase in the non-Hispanic Black/non-Hispanic White infant mortality disparity ratio. The disparity was driven largely by reduced non-Hispanic White infant mortality in high–Systemic Racism Index counties (6.5% lower per standard deviation increase), rather than increased non-Hispanic Black mortality. Key components of the Systemic Racism Index included residential segregation, incarceration rates, economic inequality, educational attainment, and employment gaps. | Systemic racism, operationalized through composite structural indicators, perpetuates racial disparities in infant mortality by embedding macro-level inequities—including segregation, mass incarceration, and economic inequality—that differentially shape population health outcomes and amplify racialized risks from birth onward. |
| Ramraj et al., 2019, USA [35] | Cross-sectional study | 15,285 mothers (Black and White) from the 1988 National Maternal and Infant Health Survey, 48 U.S. states | To determine whether Black–White infant mortality rate disparities are attributable to differences in maternal characteristics or to differential returns on those characteristics, reflecting structural racism | The Black infant mortality rate was twice that of the White infant mortality rate (18.1 vs. 8.4 deaths per 1,000 births). Differences in maternal characteristics (income, education, marital status, etc.) did not explain the disparity. Differential returns on characteristics—that is, structural barriers preventing Black mothers from translating socioeconomic advantages into improved outcomes—accounted for the majority of the disparity. | Structural racism limits Black mothers’ ability to translate socioeconomic resources into equitable infant outcomes by embedding systemic barriers across healthcare access, social support, and institutional contexts, thereby driving persistent Black–White disparities in infant mortality independent of maternal characteristics. |
| Randolph et al., 2024, USA [70] | Mixed-methods study | 44 Black cisgender women and 19 Black stylists in the Southern United States | To develop and evaluate a community-partnered, culturally relevant intervention (UPDOs) to improve pre-exposure prophylaxis (PrEP) uptake among Black women, guided by the 5Ws Racial Equity Framework | Community Advisory Councils and beauty salon partnerships improved trust and relevance. UPDOs improved PrEP knowledge, reduced stigma and mistrust, and empowered stylist-led health advocacy. The 5Ws Racial Equity Framework supported equitable research and intervention design and strengthened trust and engagement in health research. | Structural racism, through exclusion from HIV trials, medical mistrust, and inequitable resource allocation, creates barriers to HIV prevention for Black women. Community-partnered, equity-centered interventions can mitigate these barriers and foster trust in HIV prevention efforts. |
| Richardson et al., 2023, USA [81] | Qualitative study | 20 stakeholders (obstetricians/gynecologists, doulas, community organization staff, administrators) in Alabama | To identify community and system-level contributors to racial disparities in maternal health and propose solutions | Structural racism, unjust laws and policies, poverty, and inadequate infrastructure were key drivers of racial inequities in maternal outcomes. Stakeholders emphasized Medicaid expansion, community-based maternity models (eg, doulas, midwives), paid parental leave, and culturally competent care as essential strategies. | Structural racism—including restrictive Medicaid policies, punitive legal frameworks, underfunded care infrastructure, and systemic neglect—underpins racial inequities in maternal health in the Deep South by limiting access to comprehensive, equitable maternity care. |
| Riley et al., 2024, USA [31] | Cross-sectional study | 3,605,183 singleton live births in the United States in 2019 | To develop a state-level latent class measure of structural gendered racism and examine its association with preterm birth among racialized groups in the United States | Four distinct latent classes of structural gendered racism were identified, all characterized by higher levels of disadvantage for Black women and advantages for White men. Preterm birth risk was higher among Black birthing people across all classes compared to White birthing people, with some variation among Black individuals across classes. | Structural gendered racism—through intersecting systems of racism and sexism—shapes preterm birth disparities by embedding structural inequities across healthcare, labor markets, and social supports, contributing to persistent racialized and gendered health inequities. |
| Scott et al., 2023, USA [63] | Ecological cross-sectional study | 1,114 census tracts across Louisiana, USA | To analyze how residential racial and economic segregation, measured by the Index of Concentration at the Extremes (ICE), influences COVID-19 case rates | Higher COVID-19 case rates were significantly associated with racially and economically segregated neighborhoods. In Northwest/Central Louisiana, COVID-19 rates were higher in Black-concentrated areas; in Southeast Louisiana, rates were more neutral or favorable in White-concentrated areas. Geographic variation underscored structural inequality in exposure risk. | Structural racism—through residential segregation, discriminatory housing policy, and environmental inequities—drives unequal COVID-19 burdens by shaping differential exposure risk and resource access across segregated communities. |
| Santos Silva et al., 2024, Brazil [62] | Retrospective cross-sectional study | 590,102 hospitalized COVID-19 patients in Brazil in 2020, stratified by race (White, Black/Biracial, Indigenous, Others) | To examine racial disparities in COVID-19 mortality and healthcare access across public and private healthcare sectors | Black/Biracial and Indigenous patients had significantly higher mortality risk (up to 78% and 29% higher, respectively). Disparities persisted after adjustment for clinical and socioeconomic variables. Public hospitals had consistently worse outcomes. | Structural racism, via systemic underfunding of public hospitals, unequal intensive care unit access, and institutional bias, exacerbates COVID-19 mortality risks for racial minorities in Brazil’s stratified healthcare system. |
| Talbert, 2023, USA [25] | Cross-sectional study | 26,086 non-Hispanic Black adults (16,124 women; 9,962 men) from the United States, based on data from the 2017 Behavioral Risk Factor Surveillance System (BRFSS) | To examine associations between exposure to police killings (especially of unarmed Black individuals) and cardiovascular health outcomes among Black Americans | Exposure to police killings of unarmed Black individuals was associated with increased hypertension in Black women and increased stroke in Black men. Total police killings were also associated with increased stroke risk in Black men. No significant associations were found for diabetes or myocardial infarction. An inverse association with diabetes was observed in some male subgroups. | Structural racism, enacted through state-sanctioned violence and racially disproportionate policing, functions as a chronic stressor that adversely affects cardiovascular health in Black communities, contributing to racial health inequities through physiological stress pathways. |
| Thomas et al., 2020, USA [64] | Cross-sectional ecological study | 2,994 U.S. counties (COVID-19 outcomes); a subset of 957 counties with race-specific data; and 723,271 non-Hispanic White Implicit Association Test participants | To examine associations between county-level implicit and explicit anti-Black racial bias among non-Hispanic Whites and COVID-19 outcomes | Higher implicit and explicit racial bias were associated with higher COVID-19 incidence and mortality rates. Larger Black-White incidence rate disparities were observed in counties with greater racial bias. Explicit bias was a stronger predictor of disparities than implicit bias. Structural inequities such as segregation and healthcare access may interact with racial bias to exacerbate outcomes. | Structural racism, expressed through community-level racial bias and interacting with entrenched structural inequities, contributed to worse pandemic outcomes and magnified racial health disparities during COVID-19 in the United States. |
| Thomas et al., 2023, USA [73] | Qualitative study | Five Black community-based doulas and two program directors from a Medicaid-funded doula program in Los Angeles, California | To explore how Black community-based doulas address systemic racism in perinatal care and the challenges they face | Doulas encountered internalized, interpersonal, and institutional racism while supporting Black birthing clients. Racial concordance fostered trust, communication, and advocacy. Programme sustainability was challenged by lack of funding, training, and systemic support. | Structural racism directly affects both providers and patients in perinatal care. Culturally concordant doula care can buffer against systemic inequities and improve outcomes, though institutional barriers threaten the sustainability of such models. |
| Valdez et al., 2023, USA [34] | Qualitative study | Immigrant and refugee youth in Massachusetts, including seven emerging adults (aged 18–24 years) and ten youth service providers | To explore how structural racism influences sexual and reproductive health inequities among immigrant youth and to inform interventions addressing structural racism and violence | Three key themes emerged: lack of culture-centered sexual and reproductive health (SRH) supports for immigrant youth; immigration enforcement and fear restricting SRH service access; and perceived ineligibility due to legal status as a barrier to care. | Structural racism—through exclusionary immigration policies, language barriers, and culturally insensitive healthcare systems—limits access to sexual and reproductive health services for immigrant youth, exacerbating existing inequities in sexual and reproductive health outcomes. |
| Vilda et al., 2019, USA [65] | Ecological cross-sectional study | Non-Hispanic Black and White women across all 50 U.S. states and the District of Columbia (2011–2015) | To examine associations between state-level income inequality and pregnancy-related mortality by race | Higher state-level income inequality was associated with a 14–15% increase in pregnancy-related mortality among Black women, with no significant association observed among White women. Income inequality widened both absolute and relative racial disparities in pregnancy-related mortality. | Structural racism operates through state-level economic inequality to disproportionately elevate maternal mortality risk for Black women, reinforcing systemic drivers of racial inequities in maternal health. |
| Vilda et al., 2021, USA [9] | Ecological cross-sectional study | 16,469,521 live births across 1,211 U.S. counties, 2013–2017, USA (county-level, stratified by urban and rural status) | To examine associations between county-level structural racism and infant mortality across urban and rural contexts | Structural racism indicators (education, income, incarceration) were associated with 7–9% higher Black infant mortality rates and 5–6% lower White infant mortality rates in urban areas. No significant associations were observed in rural areas. | Structural racism embedded in education, justice, and income systems shapes urban-rural disparities in infant mortality, differentially affecting Black and White infant health outcomes through systemic inequities in structural determinants of health. |
| Wang et al., 2022, USA [66] | Ecological cross-sectional study | 558–565 municipalities in New Jersey, USA (March 2020–May 2021); Latinx and Black populations versus non-Latinx White populations | To assess ethnic disparities in COVID-19 infection rates and identify structural contributors | Latinx and Black communities consistently exhibited higher COVID-19 case rates across three waves. Key structural contributors included household crowding, proximity to New York City, reliance on public transportation, occupational risk, language barriers, and political ideology. Structural factors explained approximately 60% of observed disparities. | Structural racism, through mechanisms such as residential and occupational segregation, language barriers, and socioeconomic inequality, has driven disproportionate COVID-19 infection burdens among Latinx and Black populations, compounding health inequities during the pandemic. |
| West et al., 2022, USA [71] | Cross-sectional mixed-methods study | Staff representatives from 21 community-based organizations (CBOs) in Greater Boston that serve pregnant and postpartum clients of color | To identify gaps in social support services and challenges faced by community-based organizations, and explore the assets and networks used to address these gaps, with a focus on the impact of structural racism on maternal health inequities. | Structural racism, particularly historical redlining, restricts access to maternal healthcare and social support services. Housing and childcare were the most significant gaps. Lack of coordination among organizations further exacerbated disparities. | Demonstrates that structural racism manifests in healthcare and social support systems. It disproportionately affects maternal health outcomes for people of color by limiting access to housing, childcare, and quality maternal care. |
| White et al., 2023, USA [52] | Retrospective cross-sectional study | 10,874,289 inpatient delivery hospitalizations (2007–2020) across seven U.S. states | To assess racial and ethnic disparities in in-hospital maternal mortality and maternal end-organ injury and to evaluate effect modifiers. | Black women had thirty three percent higher odds of mortality or maternal end-organ injury compared to White women. Hispanic women had fourteen percent higher odds. Disparities persisted regardless of income, insurance status, or hospital characteristics. | Highlights that structural racism through healthcare access inequality, insurance disparities, residential segregation, and implicit provider bias contributes to adverse maternal outcomes. |
| Williams et al., 2018, USA [93] | Retrospective cohort study | 121,754 Black and White births (2002–2008), across 14 hospitals in 12 U.S. Hospital Referral Regions | To examine associations between racial residential segregation and stillbirth disparities. | Low and decreasing segregation reduced stillbirth risk for Black mothers. Decreasing segregation could prevent approximately nine hundred thirty two stillbirths annually among Black mothers. No significant effect was observed for White mothers. | Demonstrates that structural racism through residential segregation shapes racial disparities in stillbirth. Segregation is a modifiable determinant of health inequity for Black women. |
| Wright et al., 2022, USA [28] | Retrospective cohort study | 15,689 women diagnosed with invasive breast cancer in 28 Massachusetts municipalities with historical redlining maps (2005–2015) | To examine associations between historical redlining and contemporary racialized economic segregation with breast cancer incidence by hormone receptor status. | Breast cancer incidence varied by the interaction of historical redlining and current racialized economic segregation. The incidence of estrogen receptor negative and progesterone receptor negative breast cancer was highest in areas historically privileged but currently deprived. | Demonstrates that structural racism through both historical housing policies and current racialized economic segregation shapes present day cancer disparities through persistent neighborhood disadvantage. |
| Yang et al., 2025, USA [53] | Retrospective cohort study | 5,597 adults with rheumatic conditions residing in Massachusetts and neighboring U.S. states | To examine how historical redlining and present day racialized economic segregation influence healthcare utilization. | Individuals in historically redlined areas had higher odds of missed appointments and emergency visits. Residents in the most economically deprived neighborhoods experienced increased healthcare fragmentation. | Highlights the lasting effects of structural racism through historical redlining and contemporary economic segregation on current healthcare access and quality for patients with chronic illness. |
| Yu et al., 2024, USA [94] | Quasi-experimental study | 4,560 Medicaid-eligible birthing individuals; Kent County, Michigan | To examine whether race, ethnicity, and language concordant community health worker integrated programs improve engagement in home visiting services for high-risk birthing individuals in segregated neighborhoods. | Community health worker integrated programs improved home visiting engagement in segregated neighborhoods, reduced barriers, and provided culturally competent care. Program reach and retention were enhanced. | Demonstrates that neighborhood segregation as a manifestation of structural racism restricts healthcare access. Community health worker integrated programs can mitigate these barriers and promote more equitable healthcare engagement. |
| Yu et al., 2024, USA [74] | Retrospective cohort study | 211,412 Medicaid-eligible singleton births in Michigan, United States (2016–2019) | To assess whether home visiting programs mitigate the effects of neighborhood racialized economic polarization on birth outcomes. | Home visiting participation reduced preterm birth by 6.8 percent and low birthweight by 5.2 percent. Larger benefits were observed for Black individuals. Community health worker-integrated programs further enhanced outcomes in deprived neighborhoods. | Demonstrates that structural racism through neighborhood deprivation and racialized economic polarization contributes to adverse birth outcomes. Targeted home visiting programs can partially mitigate these structural effects, particularly for Black populations. |
| Zalla et al., 2023, USA [17] | Observational cohort study | 72,535 non-Hispanic Black and White adults entering HIV care in the United States, using data from the North American AIDS Cohort Collaboration on Research and Design (NA-ACCORD), 1996–2019 | To evaluate the impact of hypothetical clinic based interventions, including immediate initiation of antiretroviral therapy and guideline based follow up, on racial disparities in HIV related mortality. | Black patients had higher three year mortality compared to White patients. Universal antiretroviral therapy and follow up reduced the mortality gap to zero point two percent. Interventions targeting Black patients reversed the disparity. | Demonstrates that systemic racism in HIV care through delayed initiation of therapy and unequal follow up contributes to racial disparities in mortality. Supports the importance of targeted antiracist health strategies. |
| Zewdie et al., 2025, USA [13] | Observational cohort study | 380,052 non-Hispanic Black and White adults living in urban census tracts in the United States, drawn from eight nationwide cardiovascular cohorts. Cardiovascular Health Study (CHS),  Multi-Ethnic Study of Atherosclerosis (MESA),  REasons for Geographic and Racial Differences in Stroke (REGARDS),  Nurses’ Health Study (NHS),  Nurses’ Health Study II (NHSII),  Health Professionals Follow-Up Study (HPFS),  Women’s Health Initiative – Observational Study (WHI-OS),  Women’s Health Initiative – Clinical Trials (WHI-CT) | To assess associations between racial residential segregation and air pollution exposure. | Higher racial residential segregation was linked to increased exposure to fine particulate matter and nitrogen dioxide, independent of individual socioeconomic status or race. Associations persisted across cohorts. | Demonstrates that racial residential segregation as a manifestation of structural racism contributes to disproportionate environmental pollution exposure among marginalized communities. This reinforces racial health inequities. |
