# Supplementary material for: Structural racism as a fundamental cause of health inequities: a scoping review
Source: Int J Equity Health. 2025 Oct 8;24:257. doi: 10.1186/s12939-025-02644-7 (PMC12506018; doi:10.1186/s12939-025-02644-7)
Supplement: Supplementary file 2 — Supplementary Material 2. [file 12939_2025_2644_MOESM2_ESM.docx]

**Supplementary Table 2. Summary of Studies on Health Outcomes, Implemented or Proposed Interventions, and Their Effectiveness**

| **Author(s), Year, Country** | **Health Outcomes Affected** | **Interventions Implemented** | **Effectiveness** |
| --- | --- | --- | --- |
| Bassler et al., 2024, USA [41] | Time to human immunodeficiency virus viral suppression (defined as viral load <200 copies per millilitre); Neighborhood-based disparities in human immunodeficiency virus treatment outcomes. | No direct interventions were tested. The study highlights systemic barriers such as disinvestment in historically redlined areas and the limited impact of gentrification on health outcomes. | Not applicable. The findings underscore the urgency of implementing targeted health equity interventions to address structural barriers in marginalized urban communities. |
| Beyer et al., 2019, USA [58] | Higher cancer mortality rates among Black populations in metropolitan areas with high levels of mortgage discrimination; Elevated cancer incidence in Black men living in segregated areas; Overall racial disparities in cancer mortality present in 88% of metropolitan areas studied. | No intervention was tested . The study discusses the potential value of structural interventions such as housing mobility programs and anti-discrimination policies in mortgage lending. | Not evaluated. The findings highlight the importance of addressing housing discrimination and its downstream effects on cancer outcomes through structural and policy reforms. |
| Bishop-Royse et al., 2021, USA [59] | Infant mortality rates. | No direct intervention was tested. The Index of Concentration at the Extremes was used as a tool to monitor racial and economic segregation and marginalization. The study recommended a shift in focus from individual-level to community-level interventions. | Structural racism, as measured by the Index of Concentration at the Extremes, showed strong and independent associations with infant mortality rates. The study cautioned that individual-level interventions such as public health campaigns may not improve outcomes and could widen disparities if structural conditions are ignored. Structural reforms are recommended for achieving an equitable impact. |
| Bitsie et al., 2024, USA [15] | Perceived discrimination in healthcare: Potential implications for treatment adherence, delays in cancer screening, patient satisfaction, and cancer outcomes. | No intervention was tested. The study identified the need for implicit bias training, anti-racism policies, and improved cultural communication to address the discrimination reported by patients. | Intervention effectiveness was not evaluated. However, the study provides strong evidence that addressing discrimination is essential to improving the quality of cancer care and achieving health equity in oncology. |
| Boley et al., 2024, USA [20] | Disparities in pain management, specifically opioid administration and prescribing, for non-Hispanic Black and Hispanic patients presenting with abdominal pain in the emergency department. | No intervention is explicitly mentioned. The study focused on identifying disparities rather than testing interventions. | Not applicable. The study highlights existing disparities in pain management but does not evaluate interventions. The findings reinforce the need for systemic reforms in clinical practice to address racial inequities in pain treatment. |
| Brase et al., 2021, USA [42] | Preterm delivery (defined as delivery before 37 weeks of gestation). | Statistical adjustment for known sociodemographic, pregnancy-related, and preconception risk factors, including body mass index, access to prenatal care, birth interval, and marital status. | Adjustment reduced the preterm delivery disparity gap from 77% to 47%. Substantial inequity remained after adjustment, indicating that unmeasured structural and life-course factors linked to racism contribute to persistent disparities in preterm birth outcomes. |
| Canales et al., 2023, USA [43] | All-cause mortality; Breast cancer-specific mortality. | No intervention was tested. The study highlighted disparities in mortality linked to residential segregation. Structural interventions were suggested to address resource inequities associated with segregation while preserving community-level protective factors. | Not applicable. The study identified mortality disparities but did not test interventions. Findings underscore the need for structural reforms targeting segregation-related inequalities in health resources. |
| Cascino et al., 2022, USA [14] | Lower rates of ventricular assist device implantation and heart transplant among Black patients; Higher all-cause mortality (18% in Black patients compared to 13% in White patients). | Multivariable adjustment for heart failure severity, patient preferences, and social determinants of health. The study proposed reforms, including implicit bias training and standardization of psychosocial evaluation procedures. | Adjusting for clinical and social factors attenuated, but did not eliminate, disparities. The findings highlight that targeted antiracist reforms in care delivery processes are necessary to achieve equitable access to life-saving treatments such as ventricular assist devices and transplants. |
| Chambers et al., 2019 (2018), USA [82] | Preterm birth (9.6% incidence); Infant mortality (5.4 deaths per 1000 live births, or 0.5%). | No direct intervention was tested. The Index of Concentration at the Extremes was used as a tool to monitor racial and economic segregation and marginalization. | The Race plus Income Index of Concentration at the Extremes score showed the strongest associations: adjusted odds ratio 1.25 for preterm birth and 1.68 for infant mortality. The study validated the Index of Concentration at the Extremes as a structural racism indicator and risk-monitoring tool in maternal and infant health outcomes. |
| Chan et al., 2024, USA [44] | Pregestational diabetes (relative risk 1.7 in neighborhoods with Grade D historical redlining); Chronic hypertension (relative risk 1.5); Gestational diabetes; Hypertensive disorders of pregnancy; Small-for-gestational-age births; Low birthweight. | No intervention was tested. The study provided evidence to support the need for targeted preventive care in historically marginalized areas affected by redlining and ongoing structural inequities. | Not applicable. The study did not evaluate interventions but demonstrated significant associations between historical redlining and adverse maternal and birth outcomes, highlighting the need for structural preventive strategies. |
| Chegwin et al., 2023, USA [83] | Low birth weight and preterm birth among Black women. | No direct intervention was tested. The analysis focused on exposure to racialized police use of force at the community level. The study implied the need for structural policing reforms, including antiracist community policing policies. | Not applicable. The study found strong associations between racialized structural violence and adverse birth outcomes in Black women, reinforcing the importance of structural changes in public safety and health policy to improve maternal and infant outcomes. |
| Chen et al., 2022, USA [84] | Youth psychiatric emergencies, including suicidal ideation, self-harm, and behavioral crises; Disproportionate use of mobile crisis response services among Black and Multiracial youth. | Mobile crisis response services are provided by the Los Angeles County Department of Mental Health; Public mental health safety-net services. | Mobile crisis response services provided rapid crisis response but may reflect systemic biases in crisis detection and service pathways. No direct intervention evaluation was conducted. Findings indicate an urgent need for more equitable access to mental health care and the development of antiracist crisis response policies to address disparities in youth psychiatric emergencies. |
| Collin et al., 2021, USA [11] | Breast cancer mortality. | No interventions were implemented. The study identified historical redlining as a modifiable structural factor and suggested housing policy reforms to address structural drivers of health disparities. | Not applicable. The study did not test interventions but identified the legacy of redlining as a driver of breast cancer mortality disparities. Findings support the need for place-based interventions targeting housing inequities and community-level resources. |
| Daoud et al., 2022, Israel [75] | Maternal psychological distress, including feelings of inferiority and perceived discrimination; Inequitable postpartum care; Emotional harm and mistrust in healthcare. | Hospital policies nominally prohibited racial maternal separation but lacked monitoring and enforcement; Cultural sensitivity training, often misused to justify separation practices; Market-based approaches offering private rooms to reduce interpersonal conflict. | Existing interventions were largely ineffective. Racial maternal separation practices continued under economic and political pressures. Cultural sensitivity training was misapplied, reinforcing segregation. Market-based solutions reduced visible interpersonal conflict but perpetuated systemic racism and inequity in maternal care experiences. |
| Davis et al., 2023, USA [60] | Preterm birth; Low birthweight. | No direct interventions were tested. The study analyzed structural exposure to municipal reliance on fees and fines levied through the criminal legal system. | Reliance on court-imposed and police-imposed fines and fees was a statistically significant risk factor for adverse birth outcomes. Results support the need for structural-level public health and fiscal reforms to mitigate the health harms caused by racially inequitable municipal revenue practices. |
| English et al., 2024, USA [29] | Suicidal ideation; Suicide attempts. | Proposed interventions included repealing racist and heterosexist state laws, such as discriminatory policing laws and HIV criminalization statutes, and enacting federal protections such as the Equality Act. | No direct interventions were tested. Statistical modelling demonstrated that repealing discriminatory policies and reducing policing exposure could reduce suicidality among Black lesbian, gay, bisexual, queer individuals. The study highlights the need for structural policy interventions rather than solely individual-level suicide prevention approaches. |
| Francis et al., 2023, USA [76] | Hypertensive disorders of pregnancy, maternal stress, poor nutrition and food insecurity, anxiety and depression, missed prenatal care, limited physical activity | No interventions were tested. Policy recommendations included the Black Maternal Health Momnibus Act, infrastructure investment, clinician training on anti-racism, and community resource expansion. | Effectiveness not directly evaluated. Findings support the need for multi-level, structural policy responses that simultaneously target intersecting domains of racism rather than siloed solutions. |
| Gadela et al., 2022, USA [27] | Pediatric asthma mortality and morbidity | No specific interventions were tested. The study highlights the importance of multifactorial interventions addressing individual, environmental, and societal risk factors. | No intervention evaluated. Findings underscore the need for community-driven initiatives and structural interventions to address racism and improve pediatric asthma outcomes. |
| Gao et al., 2024, USA [10] | Severe maternal morbidity and increased maternal mortality risk among racially marginalized groups | No formal interventions were tested. Findings suggest that addressing structural drivers of adverse pregnancy outcomes, such as housing policies and systemic reforms, could mitigate disparities. | No intervention evaluated. Results highlight the need for targeted housing and anti-displacement policies as key strategies to mitigate structural racism’s impact on maternal health. |
| Guglielminotti et al., 2024, USA [54] | Severe adverse maternal outcomes, including eclampsia, blood transfusion, hysterectomy, and intensive care unit admission | No interventions were implemented. The study discusses potential policy implications for addressing structural racism as a driver of maternal health disparities. | No intervention evaluated. The study focuses on identifying associations between structural racism indicators and maternal outcomes, underscoring the need for systemic policy reforms. |
| Hailu et al., 2024, USA [23] | Severe maternal morbidity, defined as life-threatening complications during childbirth (e.g., eclampsia, heart failure) | No interventions were tested. The study suggests community-based alternatives to punitive criminal-legal practices as a means to mitigate health impacts. | No intervention evaluated. The study emphasizes the need for systemic interventions to address incarceration inequities as drivers of maternal morbidity disparities. |
| Harville et al., 2022, USA [68] | Low birthweight and preterm birth | No specific interventions were tested. The study recommends legal assistance, financial counseling, and expanded public housing availability as potential policy solutions. | No intervention evaluated. Findings reinforce the importance of addressing housing instability through structural interventions to improve maternal and infant health outcomes. |
| Havens et al., 2011, USA [77] | Racial disparities in healthcare access, quality, and outcomes; organizational barriers to equity in service delivery | Mandatory Dismantling Racism training, voluntary caucus groups, equity initiatives led by Change Teams | Increased racial equity awareness among participants; some reported improvements in organizational policies and practices; mixed responses with some staff reporting workplace tension; effectiveness linked to leadership engagement and cultural fit. |
| OjiNjideka Hemphill  et al., 2023, USA [19] | Maternal morbidity, severe pregnancy complications, increased cesarean section rates, medical mistrust, pregnancy-related trauma | No formal intervention was tested. Participants relied on advocates (e.g., family) and trusted healthcare providers to navigate care; the study highlights the need for systemic change. | Advocacy and trusted provider relationships improved participants' autonomy and reduced mistrust. Systemic healthcare reforms are required to address underlying structural racism in maternity care. |
| Henderson and Quenby, 2025, UK [85] | Preterm birth, small-for-gestational-age outcomes | Analysis of latent socioeconomic position and antenatal access; adjustment for income, education, and marital status | Latent socioeconomic position explained up to 60% of preterm birth disparities and 53% of small-for-gestational-age disparities, whereas traditional measures explained only 10–29%. No intervention tested; findings call for structural social policy reforms. |
| Hernandez et al., 2024, USA [86] | Screening mammography uptake, breast cancer stage at diagnosis | No direct intervention was tested; the study measured the impact of perceived discrimination, trust, and neighborhood deprivation on outcomes | Higher perceived discrimination reduced the likelihood of screening (adjusted odds ratio 0.956) and increased the risk of late-stage diagnosis (adjusted odds ratio 1.062). Highlights the need for interventions to address discrimination and build trust in healthcare settings. |
| Hollenbach et al., 2021, USA [45] | Preterm birth, extreme preterm birth, periviable birth, severe maternal depression, substance use disorder, pregnancy-related hypertension, low APGAR scores, neonatal intensive care unit admissions, lower breastfeeding rates | No intervention was tested; historical redlining analyzed as structural exposure | Not applicable. The study underscores the legacy effects of structural racism and calls for policy-level interventions to redress health inequities rooted in historical disinvestment. |
| Homan and Brown, 2022, USA [33] | Depressive symptoms, functional limitations, limitations in instrumental and basic activities of daily living among older Black adults | No direct interventions were tested. Policy reforms proposed to reduce racialized felony disenfranchisement, including restoring voting rights and expanding access to political participation. | Not empirically tested. Findings suggest that addressing felony disenfranchisement as structural racism could help reduce racial health disparities and improve population health equity. |
| Hung et al., 2022, USA [55] | Severe maternal morbidity | No specific interventions were tested; study recommends policy reforms to reduce residential segregation, improve healthcare access, and address structural racism. | No interventions evaluated. Findings highlight the need for targeted structural policies and community-based interventions to mitigate severe maternal morbidity disparities linked to structural racism. |
| Hunte et al., 2022, USA [78] | Maternal and infant health outcomes, including maternal stress, preterm birth, and maternal mortality (qualitative findings) | Culturally specific care through Healthy Birth Initiatives, racial concordance between providers and clients, a reproductive justice framework, and community-based holistic support | Improved trust, emotional safety, and engagement among clients; empowered both providers and clients. Effectiveness constrained by underfunding and systemic bias in public health systems; findings highlight the need for sustained support for culturally specific, equity-driven programs. |
| Igbinosa et al., 2023, USA [87] | Severe maternal morbidity, nontransfusion severe maternal morbidity (organ failure, intensive care unit admission), preterm birth, hemorrhage, stillbirth, racial disparities in anemia prevalence | Antepartum anemia screening and management. Multivariable adjustment for social determinants and delivery factors. Calls for equitable anemia screening guidelines, elimination of race-based thresholds, and universal treatment protocols. | Adjusting for anemia reduced the risk of severe maternal morbidity across racial and ethnic groups. Anemia explained approximately 20% of severe maternal morbidity disparities for Black, Hispanic, and multiracial patients. Systemic changes in screening and treatment could reduce disparities, though no formal intervention trial was conducted. |
| Jadow et al., 2023, USA [12] | Stroke prevalence, particularly in historically redlined Black and Hispanic communities | No interventions were tested. The study analyzed the policy legacies of redlining and modern structural factors such as poverty, education, and healthcare access. | Not applicable. The study showed that structural racism through redlining continues to shape geographic disparities in stroke prevalence and called for structural reforms. |
| Jahn et al., 2020, USA [22] | Preterm birth, particularly elevated risk for Black women and in counties with high incarceration rates | No direct interventions were tested. Suggested policies include reducing jail incarceration, such as bail reform and alternatives to arrest. | Not directly tested. Racial disparities in preterm birth decreased after adjusting for Black–White incarceration disparities. The study suggests that reducing incarceration could mitigate preterm birth risks and narrow racial inequities. |
| Jahn et al., 2021, USA [24] | Pregnancy loss, proxied by reductions in live births. Racial disparities in birth outcomes are linked to exposure to police violence. | No direct intervention was tested. The study analyzed exposure to fatal police violence as a structural factor. | Statistically significant association found between fatal police violence and decreased live births, particularly for Black women (minus 0.29% per fatal event during gestation). Study underscores need for systemic interventions addressing structural racism in policing. |
| Jahn et al., 2023, USA [7] | Preterm birth, a leading cause of infant mortality, disproportionately affecting Black birthing people | No direct intervention was tested. The study suggests reducing proactive policing and promoting community-driven alternatives to public safety to mitigate racial health disparities. | Not applicable. The study did not evaluate interventions but highlighted the urgent need for structural and policy reforms to dismantle racialized policing and its impact on Black maternal health. |
| James and Horne, 2024, USA [88] | Increased internalized racism is linked to lower healthcare-seeking attitudes and disparities in mental, medical, vision, and dental healthcare utilization | No formal interventions were tested. The study suggests removing structural barriers to healthcare access (such as improving insurance coverage, reducing transportation barriers, combating discrimination), and addressing internalized racism to improve healthcare-seeking behaviors. | No direct interventions were evaluated. The study suggests that addressing structural barriers and internalized racism could improve healthcare-seeking attitudes and behaviors across healthcare domains. |
| Janevic et al., 2025, USA [32] | Postpartum hypertension, elevated systolic and diastolic blood pressure, increased risk of future cardiovascular disease | Text-based home blood pressure monitoring with telemonitoring. Engagement of a Community Working Group in study design and interpretation. | Blood pressure monitoring improved early detection of elevated blood pressure but did not mitigate the impact of gendered racial microaggressions or structural racism. No interventions directly targeting racism were tested; findings underscore the need for systemic and clinical reforms. |
| Jeffers et al., 2023, USA [69] | Severe maternal morbidity, including complications such as cardiac arrest, eclampsia, sepsis, acute renal failure, and hysterectomy | No specific interventions were tested. The study highlights systemic inequities in healthcare access and quality as key contributors to poor outcomes. | Not applicable. The study did not evaluate interventions but underscores the need for structural policy changes addressing economic segregation and healthcare disparities to improve maternal health outcomes for Black women. |
| Karvonen et al., 2022, USA [47] | Frequent acute care use, hospital readmissions, and post-discharge infant mortality | No interventions were directly tested. Study advocates for using measures of structural racism, such as racial and economic segregation indices, for identifying health inequities and proposes redistributive and antiracist policy reforms. | Structural racism remained a strong predictor of adverse outcomes. Adjusting for social determinants (such as nutrition program enrollment, education, and insurance) reduced disparities but did not eliminate them, highlighting the need for structural reforms. |
| Karvonen et al., 2025, USA [46] | Lower five-year and ten-year cancer survival in adolescents and young adults. Higher mortality risk in young adults (hazard ratio 1.34) | No direct interventions were tested. Policy recommendations include housing vouchers, neighborhood reinvestment, and expansion of Medicaid and the Children’s Health Insurance Program. | Not evaluated. The study highlights the need for structural policy interventions to address the ongoing effects of historical redlining on cancer survival outcomes among marginalized youth. |
| Khanijahani and Tomassoni, 2022, USA [61] | COVID-19 mortality, with disproportionate burden in counties with high residential racial and socioeconomic segregation | No interventions tested. The study identified structural drivers of disparities. | Not applicable. The study calls for multi-level policies targeting segregated neighborhoods and addressing structural determinants of COVID-19 disparities. Recommends explicitly considering segregation in pandemic preparedness and response policies. |
| Lee et al., 2024, USA [89] | Increased risk of hypertensive disorders of pregnancy, maternal health disparities in pregnancy outcomes, stress-related health complications due to racism and discrimination | No direct interventions were tested. The study analyzed the impact of racism and discrimination on hypertensive disorders of pregnancy. Findings suggest that systemic racism influences disparities in maternal health outcomes. | Not applicable. The study did not evaluate specific interventions. However, racial disparities in hypertensive disorders of pregnancy were reduced when perceived experiences of racism and discrimination were included in the analysis. The study highlights the need for addressing racism in healthcare to improve maternal outcomes. |
| Li et al., 2024, USA [48] | Reduced access to live donor kidney transplantation for Black individuals, persistent racial disparities in transplant outcomes | Suggested interventions include community outreach clinics, policies addressing neighborhood investment, diversifying transplant center leadership, and reforming transplant metrics to promote equity. | Not applicable. No direct interventions were tested. The study underscores the need for targeted outreach and equity-focused policies to address the impacts of segregation on access to live donor kidney transplantation. |
| Lubarsky et al., 2024, USA [16] | Receipt of guideline-concordant treatment for breast cancer, stage at diagnosis, tumor grade (with poorer differentiation among non-Hispanic Black women) | No interventions were tested. The study advocates for structural interventions to improve equity in cancer care delivery. | Not applicable. The study was observational but strongly supports the need for anti-racist, equity-focused reforms in cancer care delivery. |
| Ly et al., 2023, USA [49] | Higher postoperative mortality at 7, 14, 30, and 60 days, with the greatest disparities in elective surgical mortality among Black men | No direct interventions were tested. The study suggests potential strategies, including Enhanced Recovery After Surgery protocols and standardized perioperative care pathways. | Not evaluated. Enhanced Recovery After Surgery protocols have shown promise in reducing surgical disparities in other contexts. The study recommends these interventions as potential strategies to reduce racial disparities in surgical outcomes, but effectiveness in this population was not directly tested. |
| Machado et al., 2021, Brazil [67] | Obesity incidence, particularly among Black individuals (body mass index ≥ 30 kg/m²) | No interventions were tested. The study suggests developing public policies that promote racial equity in healthcare, anti-discrimination campaigns, and socioeconomic support programs. | Not applicable. No interventions were tested. However, findings underscore the need for equity-oriented policies targeting social determinants of health and racial discrimination as modifiable risk factors for obesity. |
| Mahabir et al., 2021, Canada [79] | Mental health distress (including anxiety and distrust), delayed or avoided care, under-treatment (including pain management), unequal access and treatment adherence | Existing cultural competence initiatives were critiqued as inadequate. The study proposes anti-racist policies focused on reducing power imbalances, institutional reform, anti-racist pedagogy, and systemic accountability. | Cultural competence initiatives were found to be ineffective in addressing systemic racism. Anti-racist policy interventions were recommended but not evaluated. Structural changes were prioritized over individual-level bias training for sustainable impact. |
| Maldonado et al., 2022, USA [30] | Mental health outcomes, including anxiety, depression, post-traumatic stress disorder, and intimate partner violence perpetration | No interventions were tested. The study suggests trauma-informed care addressing racial trauma, culturally grounded therapies such as ethnopolitical therapy, and structural reforms to reduce racism. | No direct interventions were assessed. The study proposes multi-level solutions, including clinical and systemic interventions targeting racism-induced stress, to reduce the risk of intimate partner violence and improve mental health outcomes. |
| Matoba et al., 2019, USA [90] | Preterm birth (before 37 weeks of gestation) among African American mothers | No interventions were tested. The study suggests that equitable housing reforms are necessary to mitigate the effects of historical redlining on maternal and infant health. | Not applicable. It demonstrated that institutional racism through housing discrimination continues to adversely impact maternal and infant health outcomes. |
| Matthews et al., 2021, USA [18] | Postpartum depression, anxiety, birth-related trauma, and undiagnosed or untreated perinatal mental health disorders | Proposed interventions included provider education on racism and trauma-informed care, expansion of the Black women's mental health workforce, funding for Black-led community-based organizations, inclusion of traditional healing practices such as doulas and midwives, and models of integrated care and shared decision-making. | Community-based and culturally grounded approaches were found to be promising for improving trust and engagement. Structural barriers such as insufficient funding and a lack of trained providers hindered the widespread adoption and effectiveness of these interventions. The study highlights the need for sustained policy and funding support to enable long-term change. |
| McGrath et al., 2023, USA [72] | Central line-associated bloodstream infections | Stratification of infection rates by race, ethnicity, and language in dashboards; improved tracking of catheter maintenance; inclusion of family feedback and equity-related questions in infection event reviews; enhanced interpreter access; antiracism and equity training across the hospital | Significant reduction in central line-associated bloodstream infections among Black patients (−1.77 per 1000 catheter days, P = 0.03) and non-English speakers (−1.25 per 1000 catheter days, P = 0.01). Disparities narrowed post-intervention. Sustainability was affected by external factors such as COVID-19-related staffing shortages. |
| Mendez et al., 2011, USA [56] | Bacterial vaginosis, low birth weight, maternal perceived stress, and self-reported general health | Development and application of a redlining index to measure institutional racism | No interventions were implemented. The redlining index effectively captured structural inequality and racialized neighborhood conditions. Health effects were modest and indirect in statistical models. The study supports embedding institutional racism metrics into public health surveillance systems. |
| Miller-Kleinhenz et al., 2024, USA [50] | Estrogen receptor-negative breast cancer in Black women, late-stage diagnosis in White women, breast cancer-specific mortality (particularly in White women exposed to persistent mortgage discrimination) | No interventions were tested; examining the effects of historical redlining, contemporary mortgage discrimination, and persistent mortgage discrimination | Historical redlining and persistent mortgage discrimination were significantly associated with worse breast cancer outcomes. The findings underscore the need for structural-level interventions in housing and healthcare policy to reduce disparities in cancer outcomes. |
| Mohottige et al., 2023, USA [91] | Chronic kidney disease, diabetes, and hypertension | No interventions was tested; key structural racism indicators were identified, including residential segregation, violent crime, eviction rates, and education disparities | No interventions were evaluated. The study highlights how structural racism indicators are strongly associated with chronic disease prevalence and suggests that addressing these upstream social determinants is essential for improving population health equity. |
| Nardone et al., 2020, USA [57] | Preterm birth, low birth weight, small-for-gestational-age, and perinatal mortality | No direct health intervention was tested. Propensity score matching was used to adjust for historical neighborhood differences based on redlining maps | Not directly evaluated. Higher odds of adverse outcomes were found in grade C neighborhoods compared to grade B neighborhoods. Lower odds were found in grade D versus grade C neighborhoods, likely influenced by gentrification. The study suggests that targeted policy interventions are needed to address historical disinvestment and the ongoing effects of structural racism. |
| Nguyen et al., 2022, USA [92] | Access to needed care, access to a personal doctor, timely access to routine and specialty care | Collection of race and ethnicity data; equity-focused performance metrics; cultural competency training; enhanced interpreter services; enrollee engagement; cross-sector programs addressing social determinants of health | Plans with higher proportions of minority enrollees demonstrated smaller disparities. Cultural competency and community engagement showed promise in improving access. However, disparities persisted, particularly for Asian and Hispanic enrollees, indicating that broader and systemic reforms are still required. |
| Nordyke et al., 2023, USA [80] | Reduced access to healthcare; mental health challenges (depression, mistrust); chronic stress linked to systemic racism; barriers to preventive care | No interventions were explicitly implemented. Participants recommended interventions such as increasing the diversity of healthcare providers, improving language access, and addressing systemic bias within healthcare systems | No interventions were directly tested. Participant feedback suggests that systemic changes, including improving access and combating discrimination, could enhance healthcare experiences and reduce racial and ethnic disparities in health outcomes. |
| Poisson et al., 2024, USA [21] | Expanded Disability Status Score, relapse rates, hospital admissions and inpatient days, visual and motor disability | Geocoding linked patient addresses to area-level deprivation indicators (vacant housing, poverty, low income). Analysis of outcomes by race, insurance type, and language status. | No clinical intervention tested. Strong associations found between socioeconomic deprivation and worse outcomes. Public insurance and non-English language were associated with diagnostic delays and greater disability. Findings suggest the need for policies improving access to therapies and language-concordant care. |
| Poulson et al., 2021, USA [51] | Higher likelihood of advanced-stage colorectal cancer diagnosis, lower access to surgical resection, worse survival outcomes | No interventions were tested. The study proposed desegregation and structural solutions to address the root causes of disparities. | Residential segregation was significantly associated with worse colorectal cancer outcomes among Black patients. Reducing segregation improved surgical access but did not fully eliminate survival disparities. Findings support the need for comprehensive structural reforms. |
| Quinn et al., 2024, USA [26] | COVID-19 vaccination uptake, medical mistrust, depressive symptoms | No formal intervention was tested. The study suggests that community engagement and trust-building strategies could help mitigate mistrust and improve vaccination uptake. | Trust in healthcare providers was strongly associated with higher vaccination rates. Medical mistrust rooted in structural racism and violence reduced trust and vaccination uptake. The study highlights the importance of systemic reform and authentic community engagement to rebuild trust in healthcare. |
| Ramos et al., 2024, USA [8] | Infant mortality disparities between non-Hispanic Black and non-Hispanic White infants | No interventions were tested. The study used a Systemic Racism Index to quantify structural factors influencing disparities. | Not applicable. Findings showed that systemic racism indicators were significantly associated with widening infant mortality disparities. The study calls for multi-level structural interventions to address these inequities. |
| Ramraj et al., 2019, USA [35] | Infant mortality rate: Black infants had twice the mortality rate of White infants (18.1 vs. 8.4 per 1000 live births), with disparities persisting across socioeconomic strata | No interventions were tested. The study highlights the need for housing equity reforms, healthcare access equity, and anti-racist education and employment policies. | Not applicable. The findings indicate that structural—not individual-level—interventions are required to address the systemic racism driving differential returns on maternal characteristics and perpetuating infant mortality disparities. |
| Randolph et al., 2024, USA [70] | Low pre-exposure prophylaxis (PrEP) uptake, HIV disparities, medical mistrust, and stigma | UPDOs intervention: salon-based edutainment videos, PrEP Navigators, stylist-led opinion leader training. The intervention was guided by the 5Ws Racial Equity Framework and supported by Community Advisory Councils. | The intervention improved PrEP knowledge and awareness, reduced stigma and mistrust, and led to high HIV/PrEP knowledge among stylists. The intervention showed high acceptability and strong potential for real-world impact, demonstrating the effectiveness of culturally tailored community-based interventions. |
| Richardson et al., 2023, USA [81] | Severe maternal morbidity, maternal mortality, delayed postpartum care, preventable complications (e.g., cardiovascular events), and unintended pregnancies | No interventions were tested. Stakeholders recommended Medicaid expansion, community-based maternity care (doulas, midwives), paid parental leave, and healthcare infrastructure investments | Effectiveness was not evaluated in this study. However, the proposed strategies align with broader evidence suggesting that such policies improve maternal outcomes, reduce disparities, and support culturally competent community models of care. |
| Riley et al., 2024, USA [31] | Preterm birth (less than 37 weeks), extreme preterm (22–27 weeks), very preterm (28–31 weeks), moderate/late preterm (32–36 weeks) | No interventions were tested. The study focused on developing and applying a latent class measure of structural gendered racism. | Not applicable). The study found that structural gendered racism strongly influences preterm birth disparities and calls for comprehensive policy-based structural change to address these inequities. |
| Scott et al., 2023, USA [63] | COVID-19 infection rates | Federal community testing pilot programs (e.g., New Orleans, Baton Rouge). Targeted vaccination campaigns in segregated areas. | Reduced racial disparities in Southeast Louisiana. Persistent disparities remained in Northwest and Central Louisiana, particularly in highly segregated Black communities. Tailored interventions were more effective when responsive to local racial and spatial dynamics. |
| Santos Silva et al., 2024, Brazil [62] | COVID-19 mortality. Intensive care unit access disparities. Delays in care for Black, Biracial, and Indigenous patients. | Strengthening the public healthcare system. Anti-racism education in medical training. Improved racial representation in leadership roles. | Despite universal healthcare coverage, systemic disparities persisted. Public sector underfunding led to worse outcomes for minority groups. The recommendations are promising but structural reforms are needed for impact. |
| Talbert, 2023, USA [25] | Hypertension in Black women. Stroke in Black men. Inverse or null associations for diabetes and myocardial infarction. | No interventions was tested. The study suggests policy reforms to reduce police violence and community mental health and stress-buffering supports. | Interventions were not directly evaluated. Findings imply that reducing exposure to police violence could reduce cardiovascular disparities. The study emphasizes the importance of gender-specific and structural interventions to address chronic stress. |
| Thomas et al., 2020, USA [64] | Higher COVID-19 incidence and mortality rates. Larger Black-White COVID-19 incidence gaps. | No interventions was tested. Policy recommendations included promoting intergroup contact, strengthening social capital, and reducing structural inequities (e.g., healthcare access, income inequality). | Not directly evaluated. The study suggests that area-level interventions targeting the reduction of racial bias and structural inequities could help mitigate observed disparities. |
| Thomas et al., 2023, USA [73] | Elevated Black maternal mortality. Disproportionate perinatal morbidity including severe hypertension, postpartum hemorrhage, preterm birth, and cesarean birth. Mistrust in the healthcare system. | Community-based, culturally concordant doula programs. Advocacy in clinical settings. Emotional, spiritual, and informational support. Relationship-centered care empowering birthing individuals. | Improved trust and rapport between doulas and clients. Enhanced client advocacy and empowerment. Potential reductions in adverse birth outcomes. Program sustainability remains challenged by structural barriers and limited funding. |
| Valdez et al., 2023, USA [34] | Sexual and reproductive health inequities including access to education and care. | Translation of sexual and reproductive health materials into multiple languages. School-based sexual and reproductive health education and services. Community health center partnerships. | Translation alone was insufficient without cultural relevance. School-based services were helpful but underutilized due to fear and lack of awareness. Community partnerships were promising but not widely known or scaled. |
| Vilda et al., 2019, USA [65] | Pregnancy-related mortality and racial inequities in maternal health. | No interventions were tested. The study focused on systemic drivers. | Income inequality significantly increased pregnancy-related mortality among Black women but had no effect among White women. The study demonstrated disproportionate structural disadvantage for Black women and calls for structural interventions to address income inequality and structural racism. |
| Vilda et al., 2021, USA [9] | Infant mortality rates. The black infant mortality rate was 12.63 per 1,000 live births; the White infant mortality rate was 5.71 per 1,000. | None was implemented. Suggested reforms include criminal justice reform, education equity, and income redistribution. | Not directly evaluated. The study proposes that systemic policy reforms targeting structural inequities could reduce disparities in infant mortality rates. |
| Wang et al., 2022, USA [66] | Elevated COVID-19 infection rates in Latinx and Black communities. | No formal interventions were tested. Structural decomposition analysis used to identify determinants. | Structural factors such as housing crowding, concentration in essential jobs, and language barriers explained approximately 60% of the infection disparity. Findings informed public health planning by identifying structural inequities that drive infection disparities. |
| West et al., 2022, USA [71] | Maternal morbidity and mortality. Preterm birth. Low birth weight. Barriers to exclusive breastfeeding. | Community-based social support services are delivered through community-based organizations focused on housing, childcare, mental health support, and access to culturally responsive care. | No formal evaluation of effectiveness. Findings suggest that addressing structural racism, improving coordination among community-based organizations, and increasing funding could enhance service delivery and reduce disparities in maternal health outcomes. |
| White et al., 2023, USA [52] | Maternal end-organ injury (including acute kidney and heart failure, sepsis). In-hospital maternal mortality. | No formal intervention was tested. Recommendations include implementing standardized clinical protocols, implicit bias and anti-racism training, equity-focused hospital reforms, and expanding Medicaid. | Effectiveness was not evaluated. Stratified analyses show that disparities persist across healthcare contexts, underscoring the need for systemic interventions. The proposed solutions align with observed trends in outcome disparities. |
| Williams et al., 2018, USA [93] | Stillbirth at or beyond 23 weeks gestation, disproportionately affecting Black mothers. | Reduction in residential segregation was observed over time (natural decrease; policy-driven change not directly tested). Housing equity policies are implied. | Black mothers experienced a 47% to 80% reduction in stillbirth odds in low and decreasing segregation areas. No significant reduction in stillbirth risk was observed for White mothers. Structural interventions to reduce residential segregation could substantially narrow racial disparities in stillbirth outcomes. |
| Wright et al., 2022, USA [28] | Breast cancer incidence by subtype: estrogen receptor positive/progesterone receptor positive and estrogen receptor negative/progesterone receptor negative. Disparities observed by race and neighborhood deprivation. | No interventions were directly tested. The study suggests implementing policy reforms in fair housing and urban economic development, alongside health equity initiatives targeting place-based disparities. | Not applicable. Findings highlight the need for multisector interventions addressing both historical and contemporary structural racism to reduce breast cancer disparities. |
| Yang et al., 2025, USA [53] | Missed outpatient appointments. Emergency department visits. Hospital admissions. Fragmented care. | No direct interventions were tested. Suggested interventions include financial relief such as property tax relief, affordable housing policies, and structural reforms to improve healthcare access. | The study argues that multilevel structural interventions may mitigate care fragmentation and improve equity in healthcare access. |
| Yu et al., 2024, USA [94] | Disparities in perinatal health outcomes. Reduced access to perinatal care. Increased risk of adverse birth outcomes. Reduced healthcare engagement among marginalized groups. Increased psychosocial distress and mental health challenges due to neighborhood segregation and systemic racism. | Strong Beginnings program: Home visiting with community health workers partnering with nurses and social workers. Maternal Infant Health Program: Traditional home visiting without community health workers. Community health workers provided culturally competent outreach, peer support, and resource navigation. | Increased program participation in segregated neighborhoods (58.4% vs. 48.3%). Higher number of healthcare visits for community health worker participants (mean 11.9 vs. 4.4 visits). Greater retention in home visiting services. Community health workers enhanced engagement without replacing professional services. |
| Yu et al., 2024, USA [74] | Preterm birth. Low birthweight. | Statewide Medicaid-sponsored home visiting program. Community health worker-combined home visiting program (Strong Beginnings). | Medicaid-sponsored home visiting reduced preterm birth by 6.8% and low birthweight by 5.2%. Among Black individuals: 12.3% reduction in preterm birth and 11.7% reduction in low birthweight. The community health worker-combined model showed similar positive trends. Home visiting attenuated the effect of neighborhood deprivation on adverse birth outcomes. |
| Zalla et al., 2023, USA [17] | Three-year HIV mortality. Disparities in antiretroviral therapy initiation and retention in care. | Universal immediate antiretroviral therapy initiation within 14 days. Guideline-based follow-up with regular visits and biomarker monitoring. Focused interventions targeting Black patients only. | Universal interventions reduced the Black–White mortality gap by 80% (from 1.0% to 0.2%). Focused interventions eliminated the gap and reversed the disparity (Black patients had 1.4% lower mortality). Black patients benefited more (a 9% reduction compared to a 4% reduction for White patients). |
| Zewdie et al., 2025, USA [13] | Increased exposure to PM2.5 and NO2. Indirectly linked to elevated risk of cardiovascular disease, asthma, cancer, and dementia. | No direct interventions were tested. Statistical modeling and spatial analysis incorporating racial residential segregation into pollution-health models. Policy relevance emphasized. | Persistent association between racial residential segregation and higher air pollution exposure. Socioeconomic status adjustment alone did not eliminate pollution disparities. Findings underscore the need for structural interventions, not individual-level or socioeconomic status-based solutions. |
