# Supplementary material for: Structural racism as a fundamental cause of health inequities: a scoping review
Source: Int J Equity Health. 2025 Oct 8;24:257. doi: 10.1186/s12939-025-02644-7 (PMC12506018; doi:10.1186/s12939-025-02644-7)
Supplement: Supplementary file 3 — Supplementary Material 3. [file 12939_2025_2644_MOESM3_ESM.docx]

**Supplementary Table 3. Overview of Health Impacts Linked to Policy and System-Level Structural Racism and Mitigation Strategies**

| **Author(s), Year, Country** | **Policy/Structural Factor Examined** | **Impact on Health** | **Recommendations** |
| --- | --- | --- | --- |
| Bassler et al., 2024, USA [41] | Historical redlining (Home Owners’ Loan Corporation grading). Gentrification. | Individuals in redlined neighborhoods experienced longer delays in achieving HIV viral suppression. Gentrification did not offset the impact of structural barriers. | Prioritize HIV intervention strategies in historically disinvested neighborhoods. Address housing inequity and embed systemic racism mitigation into urban health and planning policies. |
| Beyer et al., 2019, USA [58] | Mortgage discrimination is measured as disparities in mortgage denial rates for Black applicants. Racial residential segregation is assessed through isolation and dissimilarity indices. | Mortgage discrimination was associated with 32% larger Black–White cancer mortality disparities. Segregation was linked to higher cancer incidence among Black men. Both housing-related structural racism factors contributed to persistent disparities in cancer incidence and survival. | Enforce fair lending practices to reduce mortgage discrimination, such as transparent "loan price tags." Expand housing mobility programs to combat segregation and promote integrated living environments. Target cancer prevention and early detection programs to high-discrimination, high-disparity metropolitan areas. |
| Bishop-Royse et al., 2021, USA [59] | Racial and economic segregation (Index of Concentration at the Extremes for race and income). Socioeconomic hardship index. Late or inadequate prenatal care. Neighborhood disinvestment. | Infant mortality rates were 2.5 to 3.6 times higher in the most racially segregated and economically marginalized neighborhoods. Inadequate prenatal care and systemic hardship further worsened outcomes. | Use the Index of Concentration at the Extremes as a tool for public health monitoring and targeting equity interventions. Invest in disinvested Black neighborhoods. Design structural reforms that directly address segregation, marginalization, and disparities in healthcare access. |
| Bitsie et al., 2024/2025, USA [15] | Racial and ethnic discrimination in patient-provider communication. Structural racism in care relationships and provider attention. | Minority patients, particularly Black women, reported higher rates of perceived discrimination. Discrimination was linked to poorer patient experiences, potential delays in care, and reduced treatment adherence. | Implement implicit bias and anti-racism training for healthcare professionals. Diversify the healthcare workforce. Monitor patient feedback systematically to identify and reduce discrimination. Promote culturally concordant care to build trust and improve outcomes. |
| Boley et al., 2024, USA [20] | Structural racism in opioid prescribing practices, including disparities in opioid administration and discharge prescriptions | Non-Hispanic Black and Hispanic patients were less likely to receive opioids during emergency department visits and at discharge, resulting in inadequate pain management and contributing to distrust in healthcare | Conduct further investigation into implicit and explicit biases in opioid prescribing. Implement systemic interventions, including provider training and policy reforms to promote equitable pain management practices |
| Brase et al., 2021, USA [42] | Structural racism influences life-course risk exposures across education, wealth, healthcare access, and preconception and pregnancy experiences | Sustained racial disparities in preterm delivery persisted even among college-educated women. Racial inequities extended beyond individual behaviors and healthcare access, reflecting the cumulative impact of systemic racism | Implement life-course antiracist policies across housing, education, and economic opportunity sectors. Address upstream structural determinants of health risk. Move beyond individual-level interventions to target the root causes of health inequity |
| Canales et al., 2023, USA [43] | Residential segregation at both the local (census tract) and metropolitan (metropolitan statistical area) levels, measured via Black isolation indices | High Black isolation was linked to increased all-cause mortality and breast cancer-specific mortality. Ethnic density within highly segregated metropolitan areas may buffer some negative effects through social support mechanisms, but structural inequities in access to care and economic deprivation remained key drivers of adverse outcomes | Promote policies that reduce harmful segregation while preserving protective aspects of ethnic enclaves, such as community cohesion and social capital. Invest in affordable housing and community land trusts. Integrate anti-segregation strategies with cancer care equity initiatives. Conduct further research to better understand the dual nature of segregation’s health effects |
| Cascino et al., 2022, USA [14] | Racial bias in provider decision-making, subjective psychosocial evaluations, and unequal access linked to social determinants of health including caregiver presence, insurance status, and educational attainment | Black patients received significantly lower access to ventricular assist devices and heart transplants despite having similar clinical profiles and preferences | Implement mandatory implicit bias training for transplant teams. Standardize psychosocial evaluations to reduce subjectivity in patient assessment. Track disparities systematically across referral and allocation stages. Embed equity experts in transplant review committees |
| Chambers et al., 2019 (2018), USA [82] | Racial and economic segregation measured via the Index of Concentration at the Extremes for race, income, and their combined effects | Concentrated deprivation increased the odds of preterm birth by 25 to 32 percent and the odds of infant mortality by 54 to 68 percent. | Adopt the Index of Concentration at the Extremes as a standard tool for monitoring geographic health disparities. Target health interventions in areas with deprived postal codes. Reform housing and maternal health policies to address systemic drivers of inequity |
| Chan et al., 2024, USA [44] | Historical redlining via Home Owners’ Loan Corporation grades; current racialized economic segregation measured using the Index of Concentration at the Extremes | Individuals in formerly redlined neighborhoods (grades C and D) experienced higher rates of chronic conditions and adverse birth outcomes. Effects persisted after adjusting for present-day segregation | Prioritize preconception and prenatal care in historically redlined communities. Address broader environmental and social determinants such as green space access, air pollution, housing quality, and economic investment |
| Chegwin et al., 2023, USA [83] | Racialized police use of force and discriminatory policing patterns in Black communities | Higher rates of low birthweight and preterm birth among Black women in areas with disproportionately high police use of force. No effects observed for White women or in areas with non-racialized use of force | Recognize racialized policing as a public health issue. Implement anti-racism training, bias accountability systems, and structural reforms in law enforcement. Prioritize research and intervention in communities with elevated exposure to racialized violence |
| Chen et al., 2022, USA [84] | Neighborhood racial segregation, Child Opportunity Index disparities; education opportunity inequality | Black and Multiracial youth in low-opportunity neighborhoods had higher Mobile Crisis Response service use. Institutional biases may contribute to the over-policing of Black youth in mental health crisis. Educational inequalities further exacerbate disparities in emergency mental health outcomes | Invest in equitable neighborhood resources, including education and economic opportunity. Reform school-based mental health gatekeeping to reduce racial bias. Expand early intervention and outpatient mental health services to reduce reliance on crisis services. Address systemic drivers of neighborhood inequality linked to structural racism |
| Collin et al., 2021, USA [11] | Historical redlining and lending bias (racial disparities in mortgage approval) | Historical redlining was associated with a 60 percent higher risk of breast cancer mortality. Lending bias was associated with a 14 percent reduction in mortality, likely reflecting privileged contexts. Non-Hispanic Black women were disproportionately exposed to redlined neighborhoods, exacerbating disparities | Enforce fair housing and lending laws. Invest in historically redlined and disadvantaged neighborhoods. Incorporate place-based social determinants of health into cancer care and broader health policy. Use redlining indices to guide targeted health equity efforts |
| Daoud et al., 2022, Israel [75] | Racial maternal separation, ethno-national residential segregation, neoliberal commodification of care, and misuse of cultural sensitivity policies | Discriminatory care practices disproportionately targeted visible minority Palestinian-Arab women. Resulted in psychological harm, internalized inferiority, and reduced trust in healthcare systems. Racial maternal separation was normalized in hospital routines | Prohibit racial maternal separation and enforce anti-discrimination policies. Redesign cultural sensitivity training to explicitly address implicit bias and structural racism. Reform hospital funding structures to prioritize equitable care over profitability |
| Davis et al., 2023, USA [60] | Use of law enforcement and court-imposed fees and fines as a municipal revenue source; structural racism quantified as a percent of own-source county revenue derived from fees and fines | Increased odds of preterm birth and low birthweight in counties with higher reliance on fees and fines for municipal revenue. Associations remained robust after controlling for race, segregation, and income | Recognize and dismantle exploitative financial policing practices. Advocate for equitable, non-punitive municipal revenue structures. Include fiscal policies in health equity and structural racism reform agendas |
| English et al., 2024, USA [29] | Racist policies (Black-White incarceration disparities); heterosexist policies (anti-LGBQ laws, HIV criminalization) | Increased police stops and discrimination raised suicidality among Black LGBQ individuals. Policies protected White heterosexuals but harmed Black LGBQ participants, demonstrating structural racism and heterosexism as drivers of health inequities | Repeal discriminatory laws at the state level. Pass federal protections such as the Equality Act. Implement community-specific suicide prevention programs for Black LGBQ people. Consider reparations and restorative policies to address the harms of structural racism and heterosexism |
| Francis et al., 2023, USA [76] | Medical discrimination by providers; environmental pollution; over-policing and surveillance; housing instability; lack of transportation and healthy food access | Avoidance of prenatal care; stress-related coping behaviors; exposure to environmental toxins; increased severity of hypertensive disorders of pregnancy; emotional distress and care fragmentation | Pass comprehensive legislation such as the Momnibus Act. Reform punitive surveillance policies. Expand Medicaid and anti-racism training for healthcare workers. Invest in transportation, housing, and healthy food access. Address structural environmental injustice |
| Gadela et al., 2022, USA [27] | Structural racism including residential segregation, under-resourced communities, and implicit bias in healthcare | Black children in urban areas experienced significantly higher asthma mortality rates compared to White children, driven by poor housing, environmental pollution, and inadequate healthcare access | Implement community-driven initiatives. Address systemic inequities in healthcare access and resources. Develop policies to reduce environmental hazards in underserved Black communities. Strengthen interventions addressing individual, environmental, and societal risk factors. Educate healthcare providers about structural racism |
| Gao et al., 2024, USA [10] | Historical redlining, contemporary gentrification, displacement, and structural racism in housing and healthcare | Redlining and gentrification were associated with higher odds of severe maternal morbidity, particularly among Black, Hispanic, and American Indian/Alaskan Native birthing individuals. Structural racism in housing policies exacerbated maternal health disparities | Implement policies to reduce displacement and improve housing stability. Expand housing affordability measures. Address the legacy of historical redlining through equitable urban planning. Develop maternal health interventions for marginalized communities. Integrate housing and healthcare policies |
| Guglielminotti et al., 2024, USA [54] | State-level structural racism indicators: Black to White unemployment ratio; Black to White incarceration ratio; Black to White education disparity ratio | Unemployment and incarceration inequities significantly increased the risk of severe adverse maternal outcomes, particularly for Black women. No significant association was found for education disparities | Address structural racism in maternal care through employment and criminal justice reforms. Expand Medicaid and safety-net programs to reduce systemic inequities |
| Hailu et al., 2024, USA [23] | County-level racial inequity in jail incarceration rates (Black to White ratio) | Higher incarceration inequity was associated with increased risk of severe maternal morbidity for Black and Hispanic birthing individuals | Transform criminal-legal policies. Invest in community health resources. Address systemic disinvestment in marginalized communities |
| Harville et al., 2022, USA [68] | Eviction as a social determinant of health and a consequence of structural racism, disproportionately affecting Black communities | Non-Hispanic Black women in high-eviction counties had significantly higher odds of low birthweight and preterm birth. Housing instability exacerbates racial disparities in maternal and infant health | Expand affordable housing policies. Increase public housing availability. Provide legal protections against eviction. Improve access to financial aid and legal assistance. Reform discriminatory housing practices. Implement community-based support programs |
| Havens et al., 2011, USA [77] | Institutional racism in hiring practices, service delivery, and internal accountability, provider bias; lack of culturally inclusive outreach | Institutional racism perpetuated disparities in healthcare delivery to communities of color. Unequal representation in staffing and decision-making. Tensions between equity goals and organizational culture | Tailor antiracism training to the local context. Engage leadership in equity work. Foster inclusive staff development. Create feedback loops and evaluation strategies to track progress. Center community inclusion and culturally responsive engagement |
| OjiNjdeka Hemphill et al., 2023, USA [19] | Obstetric racism, implicit bias, lack of provider continuity, poor patient-provider communication, and systemic healthcare inequities | Young Black women experienced trauma, mistrust, and disenfranchisement due to obstetric racism. This led to delayed or avoided care, exacerbating health disparities | Implement patient-centered care models. Increase racial concordance between patients and providers. Train providers in cultural humility and anti-racism. Expand access to doulas and community-based care |
| Henderson & Quenby, 2025, UK [85] | Socioeconomic stratification by ethnicity, disparities in access to antenatal care and health education, ethnic differences in smoking and stress factors | Marginalized ethnic groups were more likely to experience late-stage diagnoses and adverse outcomes. Traditional socioeconomic measures failed to capture the full extent of disparities | Use multidimensional socioeconomic measures (latent class models). Audit care services for cultural responsiveness. Prioritize equity in public health and social policy reforms |
| Hernandez et al., 2024, USA [86] | Structural racism via redlining, Area Deprivation Index, and healthcare discrimination; interpersonal discrimination and provider mistrust | Reduced access to cancer screening, higher odds of late-stage cancer diagnosis; mistrust in providers among minoritized patients | Implement community-engaged interventions. Address provider bias and interpersonal racism. Improve outreach and accessibility for women with high perceived discrimination and neighborhood disadvantage |
| Hollenbach et al., 2021, USA [45] | Historical redlining via 1930s–1940s Home Owners’ Loan Corporation neighborhood grading; structural disinvestment in “Hazardous” neighborhoods | Higher odds of preterm and periviable births; increased rates of maternal depression, substance use, neonatal intensive care unit admissions, and adverse neonatal outcomes in historically redlined areas | Dismantle structural inequities through place-based investment. Integrate housing policy with maternal health strategy. Acknowledge and address structural racism as a determinant of obstetric health in public health planning |
| Homan & Brown, 2022, USA [33] | Racialized felony disenfranchisement laws; political exclusion disproportionately affecting Black communities | Associated with worse mental and physical health outcomes for Black older adults due to systemic political marginalization and psychosocial stress. No health impact detected in White populations | Reform voting laws to end discriminatory disenfranchisement. Recognize inclusive voting rights as a public health intervention. Expand equitable access to political participation as part of an anti-racist health policy framework |
| Hung et al., 2022, USA [55] | Residential segregation (Black-White isolation index) as a form of structural racism, restricting access to quality maternal healthcare, and contributing to severe maternal morbidity disparities | Higher severe maternal morbidity rates among Black and Hispanic women in highly segregated areas, with disparities exacerbated by structural racism and limited healthcare access. The COVID-19 pandemic further worsened disparities, particularly for Hispanic women | Implement policy reforms to reduce residential segregation and improve access to high-quality obstetric care. Enhance healthcare systems by addressing structural racism and integrating social determinants of health into electronic health records. Support community-driven initiatives to mitigate maternal health disparities in segregated communities |
| Hunte et al., 2022, USA [78] | Structural racism within healthcare systems; underfunding of Black-led and culturally specific maternal health programs; racial bias in public health practices; undervaluation and burnout of Black providers | Perpetuates disparities in Black maternal and infant outcomes. Exacerbates stress and professional burden on Black healthcare workers. Limits the sustainability and impact of culturally specific care models | Invest equitably in culturally specific, community-led programs. Implement systemic anti-racism training for healthcare professionals. Support racial concordance models and workforce wellness. Embed reproductive justice frameworks into maternal care policy |
| Igbinosa et al. (2023), USA [87] | Race-based hemoglobin thresholds; inconsistent anemia screening and treatment; structural barriers including insurance, education, and systemic racism in access | Black patients were twice as likely to have anemia as White patients. Anemia contributed substantially to severe maternal morbidity disparities. Outdated and inequitable clinical practices worsened disparities. | Eliminate race-based diagnostic thresholds in clinical guidelines. Implement universal and equitable anemia screening and treatment protocols. Address structural racism in healthcare delivery systems. Ensure policies promote culturally responsive care and health equity. |
| Jadow et al., 2023, USA [12] | Historical redlining (1934–1968); contemporary social determinants of health including poverty, low education, and limited healthcare access | Higher stroke prevalence in historically redlined communities, even after adjusting for current social determinants of health. Redlining remained a significant explanatory factor for disparities in stroke outcomes. | Conduct further research into causal pathways linking historical redlining to modern health inequities. Integrate structural racism considerations into public health and urban planning. Develop policies aimed at redressing historical housing inequities and investing in community health infrastructure. |
| Jahn et al. (2020), USA [22] | County-level jail incarceration rates; Black-White incarceration disparities; underlying structural racism in policing, economic deprivation, and justice policies | Higher incarceration rates were associated with increased odds of preterm birth. Black women faced amplified risks due to structural racism in incarceration practices. Pathways include stress, reduced social support, and economic strain. | Reform incarceration policies, including ending cash bail, using diversion programs, and reducing arrests for minor offenses. Integrate public health and criminal justice data to track spillover health effects. Address racial inequities in incarceration to improve maternal and infant health outcomes. |
| Jahn et al., 2021, USA [24] | Fatal police violence as a racialized population stressor; law enforcement practices reflecting structural racism | Decreased live births are linked to police killings, especially among Black and Hispanic women. Disproportionate reproductive harm was not seen among White populations. | Reform or eliminate policing practices that disproportionately target racially minoritized groups. Invest in social determinants of health, such as housing and education. Integrate public health perspectives in law enforcement reform. Improve surveillance systems for tracking police violence. |
| Jahn et al., 2023, USA [7] | Proactive policing as a racialized public safety strategy; over-policing in Black communities as a driver of chronic stress and adverse birth outcomes | Black birthing people in highly policed areas faced a 41% increased risk of preterm birth. Proactive policing contributes to racialized health inequities and infant mortality. | Dismantle racist policing policies and reduce reliance on law enforcement in public health. Adopt alternative, community-led violence prevention programs. Implement structural reforms in law enforcement and healthcare systems to combat systemic racism. |
| James & Horne, 2024, USA [88] | Structural barriers to healthcare access include lack of insurance, transportation issues, and discrimination; internalized racism | Increased internalized racism and structural barriers contributed to lower healthcare-seeking attitudes and disparities in mental, medical, vision, and dental healthcare utilization. Dental healthcare barriers showed distinct patterns not fully explained by internalized racism. | Implement policies to eliminate structural barriers to healthcare access, including expanding insurance coverage, improving transportation access, and addressing discrimination. Increase culturally competent care and promote equity in healthcare delivery. Develop targeted interventions to reduce internalized racism and improve healthcare-seeking attitudes. Enhance the affordability and accessibility of healthcare services, particularly for marginalized communities. |
| Janevic et al. (2025), USA [32] | Gendered racial microaggressions in healthcare interactions; Structural Racism Effect Index measuring neighborhood deprivation and racial inequities | Gendered racial microaggressions were associated with higher postpartum blood pressure, contributing to cardiovascular risk. Combined high Structural Racism Effect Index and gendered racial microaggressions resulted in the greatest blood pressure elevations and disparities. | Implement anti-racism training and respectful care protocols in obstetric settings. Extend postpartum blood pressure monitoring beyond 12 days, particularly for high-risk populations. Address broader structural racism through reforms in housing, education, and healthcare access to mitigate systemic stressors affecting maternal health. |
| Jeffers et al., 2023, USA [69] | Racialized economic segregation (ICE Race-Income); Black-White incarceration inequality | Black birthing people in counties with high racialized economic segregation had 1.45 times higher odds of severe maternal morbidity. Incarceration inequality was not significantly associated with severe maternal morbidity. | Invest in communities experiencing economic deprivation. Improve job opportunities, affordable housing, and school funding. Provide targeted support to hospitals in deprived areas. Address systemic inequities in healthcare access and quality. |
| Karvonen et al., 2022, USA [47] | Racial and economic segregation (ICE scores); access to WIC, insurance, and prenatal care quality | Higher rates of adverse postnatal outcomes among Black preterm infants in segregated neighborhoods. | Implement antiracist healthcare reforms. Increase equitable distribution of social and healthcare resources. Enhance community-health system collaboration to reduce structural inequities. |
| Karvonen et al., 2025, USA [46] | Historical redlining (based on 1930s HOLC maps). Neighborhoods graded “D” showed the highest cancer mortality risk. Persistent neighborhood disinvestment led to modern poverty (25% of redlined areas now high poverty vs. 7% in non-redlined). | Reduced 5- and 10-year survival for adolescent and young adult cancer patients in redlined neighborhoods. No significant effect in pediatric cases, possibly due to Medicaid protections in younger children. | Incorporate structural racism indicators (such as redlining exposure) into cancer risk stratification and clinical practice. Implement housing equity policies and neighborhood reinvestment. Expand Medicaid and CHIP coverage to mitigate survival disparities among young adults. |
| Khanijahani & Tomassoni, 2022, USA [61] | Residential racial and socioeconomic segregation, measured via concentrated disadvantage and Black-concentrated census tracts | Higher COVID-19 mortality in segregated areas due to compounded structural disadvantages, including reduced healthcare access, environmental risks, and pre-existing inequities. Segregation was an independent predictor of worse COVID-19 outcomes. | Implement equitable resource allocation and pandemic preparedness policies that explicitly address segregation. Improve healthcare access and public health capacity in historically marginalized communities. Adopt housing, zoning, and economic policies to reduce residential segregation and its health impacts. |
| Lee et al., 2024, USA [89] | Structural racism and discrimination in maternal healthcare; intersectional discrimination (race, ethnicity, socioeconomic status); impact of discrimination on stress-related health outcomes | Increased risk of hypertensive disorders of pregnancy among non-Hispanic Black individuals. Reduced racial disparities in hypertensive disorders of pregnancy when racism and discrimination were included in the model. Experiences of racism and discrimination contributed to disparities in maternal health outcomes. | Eliminate racist and discriminatory practices in healthcare. Include racism and discrimination measures in maternal health assessments. Address intersectional discrimination in policy and care delivery. Expand research on the biological and psychosocial effects of racism on pregnancy outcomes. Improve culturally competent care. Integrate standardized racism and discrimination measures into maternal health research and care models. |
| Li et al., 2024, USA [48] | Residential and transplant center neighborhood segregation, measured by Theil H index | Black candidates in high-segregation neighborhoods had significantly reduced access to living donor kidney transplantation. Transplant centers in high-segregation neighborhoods had lower living donor kidney transplantation rates for all patients. | Expand community outreach to underserved and segregated areas. Increase resources for transplant centers serving highly segregated communities. Monitor and publish equity metrics in transplant programs. Diversify institutional leadership to promote equitable care. |
| Lubarsky et al., 2024, USA [16] | Structural racism (Index of Concentration at the Extremes metrics for racial and economic segregation); provider bias; access barriers (insurance, transportation) | Non-Hispanic Black patients had significantly lower odds of receiving guideline-concordant treatment. Disparities remained across segregation levels. Hispanic patients also experienced disadvantages in segregated neighborhoods. | Address structural racism in cancer care delivery. Enhance access to specialized oncology care. Monitor healthcare equity using spatial and segregation metrics. Implement anti-bias training and systemic healthcare reforms. |
| Ly et al., 2023, USA [49] | Differential access to high-quality surgeons; neighborhood-level disadvantage; structural inequities in preoperative optimization and postoperative care | Black men experienced significantly higher mortality after elective surgery. Contributing factors included limited access to experienced surgeons, poorer preoperative health due to systemic inequities, and neighborhood disadvantage. Findings reflect systemic racism in surgical care pathways and healthcare access. | Implement standardized care pathways such as Enhanced Recovery After Surgery protocols. Improve equitable referral pathways to high-quality surgeons. Address systemic biases in preoperative and postoperative care. Expand community-level supports to optimize pre-surgical health in disadvantaged populations. |
| Machado et al., 2021, Brazil [67] | Structural racism in education, income inequality, and experiences of racial discrimination | Higher obesity incidence among Black individuals, particularly those with high education or with low education, and exposure to discrimination. Education did not fully protect against obesity risk in the presence of racism. | Implement intersectional public health policies that recognize racism as a social determinant of obesity. Address workplace and healthcare discrimination. Monitor and reduce chronic stress linked to racism through social and institutional reforms. |
| Mahabir et al., 2021, Canada [79] | Biomedical model treating race as biological; cultural competence frameworks; neoliberal healthcare policies (privatization, underfunding); everyday and ideological racism in care delivery | Reinforced racial hierarchies and stereotypes (e.g., assumptions about pain tolerance). Reduced access to care (e.g., insurance coverage gaps). Increased mistrust and unmet healthcare needs among racialized populations. | Replace cultural competence frameworks with anti-racist, equity-focused approaches. Reform healthcare and medical education to frame race as a social, not biological, construct. Establish anti-racism task forces within hospitals. Prioritize structural solutions including equitable healthcare funding and policy oversight. |
| Maldonado et al., 2022, USA [30] | Racial discrimination as a form of structural racism | Associated with increased mental health issues and higher intimate partner violence perpetration, particularly among low-income Black women. | Incorporate racial trauma screening in mental health services. Develop anti-racist intimate partner violence prevention programs. Implement structural reforms addressing systemic inequities in housing, employment, and healthcare. |
| Matoba et al., 2019, USA [90] | Mortgage discrimination (redlining); racial residential segregation | Higher odds of preterm birth among residents of redlined neighborhoods, particularly in highly segregated Black communities. Redlining and segregation jointly increased risk of adverse maternal outcomes. | Use redlining indices as proxies for institutional racism in public health surveillance and research. Strengthen and enforce fair housing laws to mitigate systemic discrimination. Support equity-focused housing policy reforms to address structural contributors to adverse birth outcomes. |
| Matthews et al., 2021, USA [18] | Structural racism in clinical training, diagnostic bias, workforce representation; underfunding of Black-led organizations; fragmentation between maternal and mental health systems | Delayed or missed diagnoses; elevated rates of untreated mental health issues among Black birthing people; diminished trust in healthcare systems | Fund culturally responsive and Black-led mental health initiatives, including through federal maternal health legislation; mandate anti-racism and trauma-informed training for healthcare providers; integrate traditional healing practices into perinatal care models; prioritize policies that support community-based, culturally concordant maternal mental health care |
| McGrath et al., 2023, USA [72] | Racial and linguistic disparities in catheter care observation; inconsistent availability of interpreter services; systemic under-observation of minoritized patients in infection prevention audits; absence of formal equity review frameworks in hospital policies | Persistent disparities in central line-associated bloodstream infection rates among Black and non-English-speaking patients, despite similar clinical risk factors | Routinely stratify patient safety data by race, ethnicity, and language; implement maintenance observation equity tools; incorporate formal Equity Impact Assessment frameworks into hospital policies; expand availability of interpreter services and multilingual family education; systematically include patient and family perspectives in safety and quality review processes |
| Mendez et al., 2011, USA [56] | Mortgage discrimination and redlining; historical and ongoing housing-based institutional racism | Black women were disproportionately affected by housing discrimination. Living in redlined areas was associated with increased maternal stress, higher prevalence of bacterial vaginosis, low birthweight, and adverse neighborhood conditions; contributed to persistent racial segregation and concentrated poverty | Incorporate redlining and residential segregation indices into health equity research; include institutional racism metrics in public health and perinatal studies; reform housing policies and urban planning strategies to address neighborhood-level inequities that adversely affect maternal and perinatal health |
| Miller-Kleinhenz et al., 2024, USA [50] | Historical redlining based on Home Owners’ Loan Corporation maps; persistent mortgage discrimination; contemporary mortgage discrimination | Historical redlining was linked to a higher risk of estrogen receptor-negative breast cancer among Black women and later-stage breast cancer diagnosis among White women. Persistent mortgage discrimination significantly increased breast cancer mortality among White women. Black women showed elevated breast cancer risk irrespective of the mortgage discrimination level | Address racialized housing inequities through targeted public health and housing reforms; develop community-level, race-specific interventions focused on breast cancer prevention and early detection; integrate structural racism measures into national cancer surveillance and public health planning |
| Mohottige et al., 2023, USA [91] | Structural racism indicators including proportion of White residents (reverse-coded), economic and racial segregation, area deprivation index, violent crime rates, eviction rates, voter participation, household income, education levels, and disparities in health insurance coverage | Higher structural racism burden was associated with increased prevalence of chronic kidney disease, diabetes, and hypertension; neighborhoods with higher levels of disadvantage experienced markedly worse health outcomes | Implement structural interventions to address racial residential segregation, housing inequities, and income inequality; invest in targeted economic development; promote educational equity; expand healthcare access within disadvantaged communities |
| Nardone et al., 2020, USA [57] | Historical redlining; neighborhood-level deprivation and systemic underinvestment driven by redlining | Persistent disparities in adverse birth outcomes, including preterm birth, small-for-gestational-age birth, low birthweight, and perinatal mortality, were linked to residence in historically redlined neighborhoods. These disparities persisted decades later, demonstrating long-term impacts of structural racism. Neighbourhoods undergoing gentrification showed mixed trends in outcomes | Address the legacy of historical redlining through equitable investment in historically marginalized neighborhoods; monitor and manage health impacts of gentrification; use historical data to inform causal inference in structural racism research; prioritize anti-racist urban planning and neighborhood-based health equity strategies |
| Nguyen et al., 2022, USA [92] | Incomplete and inconsistent collection of race and ethnicity data; disparities in provider networks within Medicaid plans; lack of culturally and linguistically inclusive healthcare services | Minority enrollees reported worse experiences compared to White patients, even when enrolled in the same Medicaid plans. The largest disparities were observed for Asian and Pacific Islander enrollees, with a gap of 17.4 percentage points in access to specialty care | Improve and standardize collection of race and ethnicity data across Medicaid programs; incorporate equity-focused metrics into Medicaid quality monitoring; promote racially concordant and culturally inclusive models of care; support broader anti-racism initiatives across intersecting sectors, including housing and economic policy, that directly influence healthcare access and patient experience |
| Nordyke et al., 2023, USA [80] | Systemic racism in healthcare including institutional barriers (such as resource limitations, language barriers, cultural mismatches), personally mediated biases (such as microaggressions and discrimination), and internalized racism (such as mistrust and self-devaluation) | Reduced access to healthcare; increased stress and mental health challenges; perpetuation of health disparities; increased mistrust towards healthcare providers and the healthcare system | Implement policies to reduce racial disparities in healthcare access and promote health equity; increase culturally responsive and inclusive care; increase diversity among healthcare providers to improve representation and trust; address systemic biases within healthcare settings including discrimination and microaggressions; improve access to resources such as transportation, insurance coverage, and language support; implement structural policies to ensure equitable treatment for all communities |
| Poisson et al., 2024, USA [21] | Insurance disparities between public and private insurance; neighborhood deprivation measured by poverty, vacant housing, and low income; language barriers for non-English speakers; distance to care | Public insurance was associated with delayed diagnosis and higher relapse rates (p = 0.046); children in deprived neighborhoods had higher Expanded Disability Status Scale scores and longer hospital stays (p = 0.002); non-English-speaking patients presented with more severe visual deficits | Expand access to disease-modifying therapies for underinsured children; invest in interpreter services and culturally competent care; incorporate household-level social determinants of health into healthcare research and policy frameworks; improve community outreach and support for underserved populations |
| Poulson et al., 2021, USA [51] | Racial residential segregation measured using the Index of Dissimilarity | Black patients living in highly segregated areas experienced later-stage colorectal cancer diagnosis, reduced surgical access, and worse survival outcomes; in contrast, White patients in segregated areas experienced improved surgical access | Implement urban policies to reduce residential segregation; monitor segregation metrics as part of routine public health surveillance; enforce equitable healthcare access; consider reparative justice strategies such as housing reform to address the structural roots of observed health disparities |
| Quinn et al., 2024, USA [26] | Structural racism through exposure to neighborhood and police violence; interpersonal racism in healthcare interactions; medical mistrust as a systemic barrier | Exposure to racism and neighborhood violence increased medical mistrust, reduced COVID-19 vaccine uptake, and worsened mental health outcomes; persistent distrust in healthcare was rooted in both historical and ongoing systemic racism | Address systemic racism through structural policy changes targeting housing, policing, and public investment; promote authentic, long-term community engagement to build trust in public health and healthcare systems; implement public health strategies to reduce exposure to racialized violence and promote mental health equity |
| Ramos et al., 2024, USA [8] | Structural Racism Index composed of residential segregation, Black-White incarceration ratio, economic opportunity gap, educational attainment disparities, and unemployment disparities | Higher Structural Racism Index scores were associated with larger non-Hispanic Black to non-Hispanic White infant mortality disparities; this effect was driven by lower non-Hispanic White mortality in high-index counties, not by increased non-Hispanic Black mortality; systemic racism operates at a population level and reinforces inequities through structural channels | Conduct equity audits of local policies to identify and address domains of structural racism; implement structural reforms in housing, education, criminal justice, and employment to mitigate systemic racism’s downstream effects on infant mortality; shift public health focus from individual-level interventions towards addressing root structural causes of disparities |
| Ramraj et al., 2019, USA [35] | Structural racism affecting the benefits or "returns" on maternal socioeconomic status characteristics; institutional racism in housing, education, employment, and healthcare access; residential segregation; discrimination in healthcare; wealth accumulation gaps | Black mothers received lower health returns from socioeconomic status advantages; higher education, income, or insurance status did not reduce infant mortality rates for Black mothers as effectively as for White mothers; persistent infant mortality disparities remained despite socioeconomic status gains | Address institutional racism by enforcing fair housing and anti-segregation policies; reform healthcare delivery to reduce systemic bias; target racial wealth and education gaps through equity-focused policies; implement anti-racist policies across multiple sectors that influence social determinants of health, including housing, employment, and healthcare |
| Randolph et al., 2024, USA [70] | Historical research abuses undermining trust (such as the Tuskegee legacy); exclusion of Black women from pre-exposure prophylaxis (PrEP) research and marketing; inequitable funding for community engagement | Low participation of Black women in HIV prevention research; delayed adoption of pre-exposure prophylaxis among Black women; mistrust of healthcare and research institutions | Use the "5Ws" Racial Equity Framework to embed equity in research and interventions; fund Community Advisory Councils and community engagement activities; compensate community partners and respect their timelines; recognize and leverage trusted non-clinical community spaces (such as beauty salons) as valid and effective settings for HIV prevention and care |
| Richardson et al., 2023, USA [81] | Medicaid restrictions, including limited postpartum coverage and restricted provider choice; criminalization of substance use during pregnancy through "chemical endangerment" laws; lack of paid family leave; segregation and underinvestment in Black communities | Increased rates of severe maternal morbidity and maternal mortality, especially among Black women; fear and avoidance of healthcare due to criminalization; disruptions in continuity of care; delayed access to contraception and mental health support | Expand Medicaid coverage beyond sixty days postpartum; repeal punitive laws that criminalize pregnant individuals; implement statewide paid parental leave; support community-based maternity care infrastructure, including midwives and doulas, to improve access and trust |
| Riley et al., 2024, USA [31] | Structural gendered racism, including interconnected inequities in education, employment, poverty, homeownership, health insurance coverage, segregation, voting, political representation, and incarceration; this index captures how these systemic inequities contribute to disparities in preterm birth risk among Black birthing individuals | Higher levels of structural gendered racism were associated with increased rates of preterm birth among Black birthing individuals, particularly in states with pronounced inequities in education, employment, and housing | Implement multisectoral policies that address structural inequities across multiple domains; close racial wealth gaps; promote affordable housing; expand voting rights protections; consider reparations to mitigate the health impacts of structural gendered racism |
| Scott et al., 2023, USA [63] | Racial and economic segregation, as measured by the Index of Concentration at the Extremes; historical redlining and housing discrimination; environmental racism, including industrial pollution in "Cancer Alley" | Higher COVID-19 infection rates were observed in Black and low-income neighborhoods; disparities were driven by historic disinvestment, exposure to pollution, and gaps in healthcare access; spatial variations reflected the legacy of systemic inequities | Tailor public health interventions to reflect local patterns of racial and economic segregation; invest in environmental justice and healthcare infrastructure in marginalized communities; use geospatial tools, such as the Index of Concentration at the Extremes, to inform equitable pandemic planning and emergency response strategies |
| Santos Silva et al., 2024, Brazil [62] | Dual-tiered healthcare system with unequal funding between sectors; institutional racism in clinical protocols; financial, geographic, and cultural barriers to healthcare access | Higher COVID-19 mortality was observed among racial minority populations, particularly in the underfunded public healthcare system; persistent inequalities remained even after adjusting for socioeconomic status | Increase public sector healthcare funding; mandate the collection and publication of health data disaggregated by race; integrate anti-racism education into clinical training programs and leadership development within the healthcare system |
| Talbert, 2022, USA [25] | Exposure to police killings of unarmed Black individuals; state-level violent crime; number of police per capita; income inequality; racial composition | Exposure to police violence was associated with increased hypertension risk among Black women and increased stroke risk among Black men. No significant effects were observed for heart attack or diabetes outcomes. | Recognize police violence as a public health issue; implement police accountability reforms, including de-escalation training and independent oversight; address upstream social determinants of health, such as income inequality and racial segregation; develop gender-specific public health strategies to reduce stress-related cardiovascular risks. |
| Thomas et al., 2020, USA [64] | County-level implicit and explicit anti-Black bias (measured through Implicit Association Tests and explicit measures); structural inequities including residential segregation, healthcare access disparities, and income inequality | Higher levels of racial bias were associated with increased COVID-19 incidence and mortality, particularly affecting Black populations. Black-White incidence disparities widened in counties with higher levels of explicit racial bias. Structural inequities further exacerbated these disparities. | Promote community-level initiatives that foster intergroup contact to reduce racial bias, strengthen social capital within marginalized communities, implement policies to address structural inequities in housing, healthcare access, and income distribution, and improve the quality and scope of data collection on racial disparities to inform targeted public health interventions. |
| Thomas et al., 2023, USA [73] | Institutional racism, including unstable funding, lack of training and certification for Black-led doula programs; interpersonal racism through healthcare provider bias; internalized racism as reflected in doulas’ experiences and systemic inequalities | Limited access to culturally tailored and equitable perinatal care; emotional burden and burnout experienced by Black doulas; poorer perinatal outcomes for Black birthing individuals due to systemic exclusion from supportive care structures. | Secure stable and sustained funding for Black-led and culturally specific doula programs; provide comprehensive training and certification pathways; address healthcare provider bias through anti-racism training and accountability measures; promote structural reforms that integrate community-based doulas into mainstream maternity care systems. |
| Valdez et al., 2023, USA [34] | Immigration enforcement policies; lack of culturally centered sexual and reproductive health (SRH) education and services; precarious legal status limiting access to healthcare services | Fear of deportation and concerns related to legal status prevented immigrant youth from accessing sexual and reproductive health services. A lack of culturally relevant care further exacerbated these healthcare inequities. | Develop and implement culturally centered sexual and reproductive health programs; increase awareness of available healthcare services for immigrant youth; train healthcare providers in cultural humility and trauma-informed care; advocate for immigration reform policies that reduce fear and structural barriers to healthcare access. |
| Vilda et al., 2019, USA [65] | Income inequality, measured using the Gini coefficient; prolonged exposure to income inequality | Higher state-level income inequality was associated with increased pregnancy-related mortality among Black women. Income inequality widened racial disparities in maternal mortality. | Address upstream structural drivers of maternal mortality by implementing policies that reduce income inequality, such as equitable wage laws and wealth redistribution policies; tackle structural racism through systemic reforms across housing, education, healthcare access, and economic opportunity sectors. |
| Vilda et al., 2022, USA [9] | Racial inequities in education, income, and incarceration rates | Counties with high levels of structural racism demonstrated 7 to 9 percent higher infant mortality rates among Black populations and 5 to 6 percent lower infant mortality rates among White populations in urban settings. | Reform the criminal justice system to address racial inequities; improve access to quality education for marginalized communities; support economic redistribution policies; implement comprehensive anti-racist structural reforms across sectors to mitigate the impact of structural racism on population health outcomes. |
| Wang et al., 2022, USA [66] | Residential segregation, occupational segregation, household crowding, language barriers, public transport dependence, political ideology including voter suppression and trust in public health | Disproportionate COVID-19 infection risk was observed in Latin American and Black communities. Structural vulnerabilities compounded across successive pandemic waves, widening disparities. | Address upstream structural barriers by improving housing conditions and labor protections; expand linguistically inclusive health communication; promote equitable vaccine rollout and access to healthcare; and reform political systems that perpetuate health inequities. |
| West et al., 2022, USA [71] | Structural racism in maternal healthcare, historical redlining, lack of racial diversity in healthcare leadership, implicit bias in care delivery, and poor coordination among community-based organizations | Structural racism exacerbated maternal health disparities, especially for Black and Hispanic women, by restricting access to essential services and increasing maternal morbidity and mortality. Poor coordination among community-based organizations contributed to service gaps and increased distrust in the healthcare system. | Improve racial diversity in community-based organization leadership and staff; enhance collaboration among organizations to streamline service delivery; increase funding for social support programs; implement anti-racism training for healthcare providers; integrate social determinants of health into healthcare policies; advocate for structural reforms to address systemic inequities in maternal healthcare. |
| White et al., 2023, USA [52] | Racial composition of hospital units; disparities in Medicaid access; income inequality; hospital safety-net burden; designation as Black-serving hospital; disparities in comorbidity scores; implicit bias in clinical care, including pain management and intervention access | Black-serving hospitals had elevated maternal mortality and morbidity. Black patients were more frequently denied pain relief and timely interventions. These disparities persisted despite adjustments for income, insurance, and hospital size. | Provide increased funding and structural support for safety-net and Black-serving hospitals; mandate implicit bias and anti-racism training; expand Medicaid and insurance coverage; standardize review practices for morbidity and mortality; promote workforce diversity; improve collection and usage of race and ethnicity data in healthcare policy; require hospitals to meet equity-focused accreditation standards. |
| Williams et al., 2018, USA [93] | Residential segregation measured by dissimilarity and isolation indices | High residential segregation was associated with increased risk of stillbirth, particularly among Black mothers. Decreasing segregation improved outcomes, though disparities persisted in highly segregated settings. | Promote policies aimed at reducing residential segregation, including equitable housing initiatives and stronger enforcement of anti-discrimination laws; incorporate segregation metrics in perinatal health research and policymaking; expand research to explore segregation’s impact across diverse racial and ethnic groups. |
| Wright et al., 2022, USA [28] | Historical redlining using Home Owners’ Loan Corporation grades from the 1930s; contemporary racialized economic segregation measured through geographic indices | Breast cancer incidence patterns were linked to the combined effects of historic redlining and contemporary neighborhood deprivation. Estrogen receptor-negative and progesterone receptor-negative tumors were more prevalent in areas with historically privileged but currently deprived profiles. Disparities persisted over decades, indicating the cumulative effects of structural racism. | Address housing segregation and racialized economic inequities through urban policy reforms; promote equity-focused urban development and investment in underserved communities; encourage further place-based research on the health legacies of discriminatory housing policies. |
| Yang et al., 2025, USA [53] | Historical redlining documented through Home Owners’ Loan Corporation maps; contemporary racialized economic segregation measured by spatial concentration indices | Higher rates of missed outpatient appointments, increased use of emergency care, and fragmented healthcare were observed in racially segregated and historically redlined neighborhoods. | Reform housing policy to expand access to affordable housing; address economic segregation through targeted community investments; improve healthcare access and continuity of care in historically excluded and deprived communities. |
| Yu et al., 2024, USA [94] | Neighborhood segregation measured through the Index of Concentration at the Extremes; lack of culturally responsive care models; resource deprivation in segregated neighborhoods; role of community health workers in improving healthcare engagement | Reduced engagement in home visiting programs was observed in segregated neighborhoods, perpetuating perinatal health disparities. Community health worker-integrated programs significantly improved healthcare access and engagement in marginalized communities. | Sustain funding and policy support for community health worker-integrated home visiting programs; expand culturally responsive care models to enhance engagement; implement policies that mitigate the effects of neighborhood segregation on healthcare access; strengthen training and collaboration among community health workers and licensed professionals. |
| Yu et al., 2024, USA [74] | Neighborhood racialized economic polarization measured through combined race and income segregation indices | In deprived neighborhoods, the preterm birth rate was 15.7 percent compared to 10.3 percent in privileged neighborhoods; the rate of low birthweight was 11.4 percent versus 6.1 percent in privileged areas. Home visiting programs attenuated these disparities, with particularly significant benefits observed among Black individuals. | Expand and support home visiting programs, especially those incorporating community health workers; prioritize program implementation in racially segregated and economically deprived neighborhoods; use these interventions to address the structural determinants of adverse birth outcomes; integrate equity-focused strategies into Medicaid and public health initiatives. |
| Zalla et al., 2023, USA [17] | Systemic racism in treatment timeliness and adherence; inequitable follow-up practices | Structural barriers, including housing instability and systemic bias, contributed to a persistent racial gap in HIV mortality despite the presence of federal safety-net programs. | Implement anti-racist clinical care strategies that prioritize early antiretroviral therapy and structured follow-up for Black patients; reform policies to address upstream social determinants of health such as housing instability and discriminatory policing practices. |
| Zewdie et al., 2025, USA [13] | Racial residential segregation is driven by historical discriminatory housing policies and zoning laws | Communities with higher levels of racial residential segregation experienced greater exposure to fine particulate matter (PM2.5) and nitrogen dioxide (NO2), which persisted independently of socioeconomic status and individual race. This spatial clustering of pollution burdens increases the risk of cardiopulmonary diseases. | Explicitly incorporate racial residential segregation measures in air pollution and environmental health research; reform zoning and housing policies to reduce structural disparities in environmental exposures; promote environmental justice through targeted pollution reduction strategies in segregated areas; ensure that public health monitoring includes structural drivers beyond socioeconomic status. |
